# Structure of Tau filaments in Prion protein amyloidoses

**DOI:** 10.1007/s00401-021-02336-w

**Published:** 2021-06-14

**Authors:** Grace I. Hallinan, Md Rejaul Hoq, Manali Ghosh, Frank S. Vago, Anllely Fernandez, Holly J. Garringer, Ruben Vidal, Wen Jiang, Bernardino Ghetti

**Affiliations:** 1 Department of Pathology and Laboratory Medicine, Indiana University School of Medicine, Indianapolis, IN 46202 USA; 2 Department of Biological Sciences, Markey Center for Structural Biology, Purdue University, West Lafayette, IN 47906 USA; 3 Stark Neurosciences Research Institute, Indiana University School of Medicine, Indianapolis, IN 46202 USA

**Keywords:** GSS, PrP-CAA, Tau, APrP, Cryo-EM, Neurodegeneration

## Abstract

In human neurodegenerative diseases associated with the intracellular aggregation of Tau protein, the ordered cores of Tau filaments adopt distinct folds. Here, we analyze Tau filaments isolated from the brain of individuals affected by Prion-Protein cerebral amyloid angiopathy (PrP-CAA) with a nonsense mutation in the *PRNP* gene that leads to early termination of translation of PrP (Q160Ter or Q160X), and Gerstmann–Sträussler–Scheinker (GSS) disease, with a missense mutation in the *PRNP* gene that leads to an amino acid substitution at residue 198 (F198S) of PrP. The clinical and neuropathologic phenotypes associated with these two mutations in *PRNP* are different; however, the neuropathologic analyses of these two genetic variants have consistently shown the presence of numerous neurofibrillary tangles (NFTs) made of filamentous Tau aggregates in neurons. We report that Tau filaments in PrP-CAA (Q160X) and GSS (F198S) are composed of 3-repeat and 4-repeat Tau isoforms, having a striking similarity to NFTs in Alzheimer disease (AD). In PrP-CAA (Q160X), Tau filaments are made of both paired helical filaments (PHFs) and straight filaments (SFs), while in GSS (F198S), only PHFs were found. Mass spectrometry analyses of Tau filaments extracted from PrP-CAA (Q160X) and GSS (F198S) brains show the presence of post-translational modifications that are comparable to those seen in Tau aggregates from AD. Cryo-EM analysis reveals that the atomic models of the Tau filaments obtained from PrP-CAA (Q160X) and GSS (F198S) are identical to those of the Tau filaments from AD, and are therefore distinct from those of Pick disease, chronic traumatic encephalopathy, and corticobasal degeneration. Our data support the hypothesis that in the presence of extracellular amyloid deposits and regardless of the primary amino acid sequence of the amyloid protein, similar molecular mechanisms are at play in the formation of identical Tau filaments.

**Supplementary Information:**

The online version contains supplementary material available at 10.1007/s00401-021-02336-w.

## Introduction

Neurodegenerative diseases with Tau pathology may present with diverse clinical symptoms and have distinct neuropathologies [20]. In fact, in addition to differences in the involvement of anatomic areas and affected cell types, Tau aggregates may also differ in Tau isoform composition and the structure of the Tau filament [3, 10–12, 16, 64]. In the adult brain, alternative mRNA splicing of exons 2, 3, and 10 of the *Microtubule-Associated Protein Tau* (*MAPT*) gene yields six Tau isoforms that differ by the absence or presence of one or two acidic N-terminus inserts, and whether they contain three or four repeats (3R, 4R) of a conserved tubulin binding motif at the C-terminus [25]. All six isoforms are found in the insoluble Tau deposits of Alzheimer disease (AD) and chronic traumatic encephalopathy (CTE), whereas only 3R-Tau isoforms are found within those of Pick disease (PiD) and only 4R-Tau isoforms in the deposits of other diseases such as progressive supranuclear palsy (PSP) and corticobasal degeneration (CBD) [20, 24, 26, 27]. Using cryo-electron microscopy (cryo-EM), it has been recently determined that the fold of the core of Tau filaments in sporadic and familial AD differs from those in PiD and CBD [3, 10–12, 16, 64]. Furthermore, although neurofibrillary tangles (NFTs) in AD, CTE, and primary age-related tauopathy (PART) [6, 8, 43] incorporate all six Tau isoforms, the fold of the core of Tau filaments is identical in AD and PART and different from that in CTE [12, 16, 55]. Thus, the Alzheimer Tau fold may be found in the presence (AD) or in the absence (PART) of amyloid β (Aβ); however, the specific role of Aβ in the pathogenesis of Tau aggregation in AD remains undetermined. In addition, whether the conformation and filament fold of Tau varies in NFTs from other neurodegenerative diseases in which the primary amyloid protein deposited is not Aβ [21–23, 59, 60] has yet to be determined.

The Prion-Protein Amyloidoses are dominantly inherited diseases, associated with missense, nonsense, and insertion mutations in the *PRNP* gene [22]. The co-existence of parenchymal PrP amyloid (APrP) with intraneuronal Tau aggregates has been shown to occur consistently in association with some *PRNP* mutations [22]; however, in Gerstmann–Sträussler–Scheinker disease (GSS) associated with the P102L mutation, the neuropathologic phenotype has consistently shown present of APrP but not Tau aggregates in neurons [22]. In Prion-Protein Cerebral Amyloid Angiopathies (PrP-CAA), which are associated with nonsense mutations in the *PRNP* gene [21, 22, 37], the main histologic feature is the co-existence of numerous intraneuronal Tau aggregates with APrP in the vascular compartment. Whether the co-existence of intraneuronal Tau aggregates with intraparenchymal APrP amyloid is dependent of the conformation of the APrP found in the deposits remains to be established [20, 45].

The present work focuses on PrP-CAA with a c.478C > T mutation in *PRNP* that leads to a truncated PrP protein (p.Q160Ter or Q160X) [17, 37], and GSS with a c.593T > C mutation in *PRNP* that leads to an amino acid substitution in PrP (p.F198S) [36]. In these two forms of amyloidosis [referred herein as PrP-CAA (Q160X) and GSS (F198S)], APrP coexists with numerous Tau neurofibrillary aggregates made of 3R- and 4R-Tau isoforms, suggesting that different amyloids may share pathogenetic mechanisms leading to Tau aggregation in AD, PrP-CAA, and GSS. Importantly, the anatomical distribution of the respective amyloid protein differs significantly between PrP-CAA, GSS, and AD [21, 22].

Herein, we determined for the first time the biochemical properties and atomic structures of Tau in PrP-CAA (Q160X) and GSS (F198S). This work represents a significant step towards the demonstration of the existence of different conformers of Tau in neurodegenerative diseases with Tau pathology and our understanding of the role of amyloid in the pathogenic mechanism(s) of neurodegeneration.

## Materials and methods

### Neuropathology

Neuropathology was carried out as previously described [21, 49]. Please see Supplementary Material Methods section for detailed information regarding clinical, genetic, and neuropathologic findings. One half of the brain was fixed in formalin and the other half was frozen at − 80 °C. Hemispheric coronal slabs were selected that included areas of the frontal, insular, temporal, parietal, and occipital lobes. These were submitted for histology and immunohistochemistry. Tissue samples were also obtained from representative brain regions. Brain sections were 8-μm thick and were counterstained with hematoxylin. Thioflavin S (Th-S) was used to demonstrate the presence of amyloid deposits and neurofibrillary tangles. For immunohistochemical studies, primary antibodies (Table S2) were AT8 (Thermo Fisher Scientific MN1020, 1:300), Abeta (NAB 228); anti PrP (Ab 95–108, 1:300); HT7 (1:1000), RD3 (Merck 05–803, 1:3,000), and RD4 (Upstate, 1:100). Double immunohistochemical studies were performed using the Dako En-Vision Doublestain System (Dako) following the manufacturer’s instructions.

### Sanger DNA analysis

Genomic DNA was extracted from frozen brain tissue. Polymerase chain reaction (PCR) was performed for the amplification of the *PRNP* and *AβPP* genes as described [44, 51]. PCR products were sequenced on a CEQ 2000XL DNA analysis system (Beckman Coulter, Fullerton, CA).

### Whole-exome sequencing (WES)

Target enrichment made use of the SureSelectTX human all-exon library (V6, 58 megabase pairs; Agilent) and high-throughput sequencing was carried out using a HiSeq 4,000 (sx75 base-pair paired-end configuration; Illumina). Bioinformatics analyses were performed as described [14].

### Tau extraction

Sarkosyl-insoluble Tau was extracted from gray matter of fresh-frozen frontal cortex as previously described [26]. Briefly, 6 g of tissues were homogenized in A68 extraction buffer consisting of 10 mM Tris–HCl, pH 7.4, 0.8 M NaCl, 1 mM EGTA, 5 mM EDTA, and 10% sucrose with protease and phosphatase inhibitors. After a centrifugation at 20,000 × g, the supernatants were brought to 1% sarkosyl and incubated at room temperature (RT) while shaking. The supernatants were spun at 100,000 × g for 1 h at 4 °C, and the sarkosyl-insoluble pellets were resuspended in 10 µl/g tissue 50 mM Tris–HCl, pH 7.4. This resuspended pellet was further purified by 30-fold dilution in A68 extraction buffer, followed by centrifugation at 20,000 × g for 30 min at 4 °C. The pellet, containing large contaminants, was discarded, and the supernatant was centrifuged at 100,000 × g for 1 h at 4 °C. The final pellet was resuspended in 20 mM Tris–HCl, pH 7.4, and 100 mM NaCl, and stored at 4 °C.

### Western blotting and dot-blot

For Western blotting, samples were resolved on 10% Tris–glycine gels (Bio-Rad) and blocked in 5% milk in TBS plus 0.1% Tween 20. For dot blots, 2 µl of diluted samples were dotted and absorbed onto 0.22 µm pore size nitrocellulose membranes (Santa Cruz), and then blocked in 5% milk in TBS plus 0.1% Tween 20. Primary antibodies were diluted in TBS plus 0.1% Tween 20 at the following dilutions: AT8 (Thermo Fisher, 1:1000), HT7 (Thermo Fisher, 1:1000), BR133, BR134 and BR135 (1:4000), RD3 and RD4 (Millipore, 1:1000), and 3F4 (Millipore, 1:1000).

### Immuno-electron microscopy (EM)

Immunogold labeling was carried out as previously described [26]. Briefly, a 1 µl droplet of 1:50 insoluble tau fraction diluted in PBS was pipetted onto carbon nickel TEM grids (300 mesh, Ted Pella) and blotted dry after 2 min. The grid was blocked in 0.1% gelatin in PBS for 20 min, and then, excess solution blotted off with filter paper. Primary antibody diluted 1:50 in 0.1% gelatin in PBS was added for 1 h at RT. Grids were washed three times in 0.1% gelatin in PBS for 5 min each wash. Grids were then incubated in secondary antibody diluted 1:40 in 0.1% gelatin in PBS for 1 h at RT. Secondary antibodies used were 6 nm anti-mouse immunogold particles or 10 nm anti-rabbit immunogold particles (Electron Microscopy Sciences). Secondary antibody was washed off 3 times in 0.1% gelatin in PBS and blotted dry. Negative staining was performed with NanoVan (Ted Pella) for 5 s at RT. Pronase treatment of fibrils was carried out as described [26]. Briefly, a 5 µl solution of 0.4 mg/ml pronase was dropped onto the grid, and incubated for 5 min at RT. The pronase solution was wicked off with filter paper, and then, 0.1% gelatin solution was added to the grid for blocking. Primary and secondary antibody incubations were carried out as described above. Images were taken on a Tecnai G2 Spirit Twin scope equipped with an AMT CCD Camera.

### Tau seeding assay

Tau seeds were prepared from fresh-frozen brain tissue of AD Case 2, GSS (F198S) Case 2, and PrP-CAA (Q160X) case. Tissue was thawed and homogenized in 9 volumes of buffer consisting of 10 mM Tris–HCl, pH7.4, 0.8 M NaCl, and 0.1% sarkosyl, with protease and phosphatase inhibitors. Following a 10 min spin at 10,000×*g* at 4 °C, the supernatants were brought to 1% sarkosyl and incubated for 1 h at RT while shaking. The supernatants were spun at 300,000×*g* for 1 h, and the sarkosyl-insoluble pellets were resuspended in 0.1 ml PBS/gram tissue. The resuspended pellets were sonicated with a probe sonicator at 75 W, for a total of 25 × 500 ms pulses. The probe was cleaned with isopropanol and water between samples. This resuspended pellet was then centrifuged at 100,000×*g* for 30 min at 4 °C. The pellet was resuspended in 20 μl/gram tissue and sonicated again as before. Following a 10 min centrifugation at 10,000×*g* at 4 °C, the supernatant was removed and stored at –80 °C until used as seeds on cells. HEK 293 T cells stably expressing the aggregation prone repeat domain of Tau with the disease associated P301S mutation, fused at the C-terminus to either CFP or YFP, were obtained from ATCC [33]. Cells were cultured in DMEM supplemented with 10% FBS, 1% pen/strep, and 1% GlutaMax (Invitrogen). Cells were plated at a density of 50,000 cells/well in a 12-well plate, onto coverslips treated with 0.1 mg/ml poly-d-lysine for fixed cell imaging. Cells were incubated overnight at 37 °C and 5% CO_2_. The following day, 1 µl seed material was combined with Lipofectamine 2000 (Thermo Fisher) and OptiMem medium, and incubated for 20 min at room temp. This transduction complex was then added onto the biosensor cells. Cells were incubated as before for 24–48 h. Cells were then washed with 1X PBS, fixed in 4% paraformaldehyde in PBS, and mounted onto microscope slides for imaging. Images were merged and cropped using ImageJ [53].

### Mass spectrometry sample preparation

8 M urea, 50 mM Tris–HCl pH 8.5 (100 µl) was added to 20 µl of insoluble Tau fractions. The sample was reduced with 5 mM Tris(2-carboxyethyl)phosphine hydrochloride (TCEP) and alkylated with 10 mM chloroacetamide (CAM). The sample was diluted with 100 mM Tris–HCl to a final urea concentration of 2 M and digested overnight with 2 µg Trypsin/Lys-C Mix Mass Spectrometry (1:100 protease:substrate ratio, Promega Corporation). Peptides were desalted on a 50 mg Sep-Pak® Vac (Waters Corporation) employing a vacuum manifold (Waters Corporation). After elution from the column in 70% acetonitrile, 0.1% formic acid (FA), peptides were dried by speed vacuum, resuspended in 40 µL of 0.1% FA, and filtered through a 0.2 µm spin filter (Millipore).

### Nano-LC–MS/MS analysis

Nano-LC–MS/MS analyses were performed on an EASY-nLC HPLC system (Thermo Fisher Scientific) coupled to an Orbitrap Fusion Lumos mass spectrometer (Thermo Fisher Scientific). One quarter of the sample was loaded onto a reversed phase PepMapTM RSLC C18 column (2 μm, 100 Å, 75 μm × 25 cm) with Easy-Spray tip at 450 nl/min. Peptides were eluted from 3 to 30% B over 85 min, 30 to 80% B over 3 min, 80 to 4% B for 4 min (Mobile phases A: 0.1% FA, water; B: 0.1% FA, 80% Acetonitrile). Mass spectrometer settings include capillary temperature of 300 °C and ion spray voltage was kept at 1.9 kV. Replicate injections were run with the same LC and source methods (CID mode and EtHCD/HCD mode). For the CID method, the mass spectrometer method was operated in positive ion with a 3 s cycle time data-dependent acquisition method with advanced peak determination and Easy-IC (internal calibrant). Precursor scans (m/z 400–1500) were done with an Orbitrap resolution of 120,000, RF lens% 30, maximum inject time 50 ms, 4e5 normalized AGC target, including charges of 2–7 for fragmentation with an intensity threshold of 5e3, and 60 s dynamic exclusion. CID MS2 scans were performed in the ion trap at rapid speed with a 1.6 m/z isolation window, 35% normalized CID collision energy, 2e3 AGC target, and 35 ms maximum IT. The same precursor scan settings were used for the DDA EtHCD/HCD decision tree method, but with a minimum intensity filter of 1e5. The decision tree consisted of either HCD assisted ETD or HCD (30%) activation of charge states, 3, 4, 5, and 6–8 with 2 m/z isolation window, 35 ms max IT, and 1e4 AGC target in rapid ion trap mode. The data were recorded using Thermo Fisher Scientific Xcalibur software (Thermo Fisher Scientific Inc. 2017). Data were analyzed using PEAKS X + Studio [58], including all PTMs and mutations in SPIDER search.

### Negative staining for cryo-electron microscopy

Negative stain embedded imaging of Tau fibrils was carried out by applying 3 µl of sample on affinity materials coated lacey carbon grid and incubated for 30 min, then washed 5 times with DI water, followed by addition of 1% uranyl acetate (UA). Then, UA was washed, blotted out and the grid was dried in air for 4–5 min. All negatively stained images were collected on a Tecnai-12 electron microscope.

### Graphene oxide coating on Grid

A pyrene solution was used to cover the entire TEM grid surface with a single layer of graphene oxide. This was achieved using a two-step sequence of pyrene solution coating (3 µl of 0.1 mg/ml) that was applied to the dull side of a lacey TEM grid (Ted Pella #01824) followed by 3 µl of 0.1 mg/ml graphene oxide that was applied just before the pyrene solution dried out. The grid was then incubated for 2 min before the dull side of the grid was washed three times with DI water and the shiny side was washed one time with a 3 µl droplet of DI water before drying and storing the hydrophilic grids for pyrene-mediated antibody coating.

### Affinity capture and grid freezing

Grids for cryo-EM were prepared using the Affinity Grid technique. A multi-arm Pyrene-PEG-Antibody conjugate was synthesized. Then, a 4 µl solution of Pyrene-PEG-Antibody was applied on a GO-coated grid, incubated for 5 min, and washed three times with Tris buffer. 2.5 µl of Tau sample was applied on the antibody coated side of the grid and incubated for 30 min, and washed 5 times with Tris buffer. The final volume of liquid on the grid was adjusted to ~ 3 µl before blotting for 3 s, followed by plunge freezing into liquid ethane on a cryo-plunger 3 (Gatan) in a biosafety hood.

### High-resolution cryo-EM imaging

High-resolution cryo-EM images were collected on an FEI Titan Krios at 300 kV with a Gatan K3- detector in super-resolution mode. The inelastically scattered electrons were removed using a Gatan quantum energy filter with 20 eV slit width. For PrP-CAA (Q160X), we recorded 55 movie frames with an exposure time of 62 ms/frame, with a dose rate of 1.067 electrons per Å^2^ per frame for a total accumulated dose of 53.35 electrons per Å^2^ at a pixel size of 1.078 Å. For GSS (F198S), we recorded 50 movie frames with an exposure time of 62 ms/frame, and a dose rate of 1.067 electrons per Å^2^ per frame for a total accumulated dose of 53.35 electrons per Å^2^ at a pixel size of 1.078 Å. The datasets for PrP-CAA (Q160X) Tau and GSS (F198S) Tau are composed of 2004 and 1920 micrographs, respectively, with defocus values ranging from − 0.5 to − 2.5 μm. Corrections of super-resolution frames were done for gain reference, binned by a factor of 2, and then, frames were motion-corrected and dose-weighted using MotionCor2 [65]. The contrast transfer function (CTF) of all aligned and non-dose-weighted micrographs were estimated using Gctf [63].

### Helical reconstruction

All subsequent image-processing was performed in RELION 3.1 software [31, 52]. Particles were picked manually using RELION helical picker as end-to-end segments. We extracted all the segments with a box size of 768 pixel (827 Å) to cover complete crossover, down scaled to 256 pixels to speed up analysis and an inter-box distance of ~ 10% of the box length. Several rounds of reference-free 2D classifications were carried out to find homogeneous subsets and remove junk particles using a regularization value of *T* = 1–2. PHFs and SFs in PrP-CAA (Q160X) were visually identified from the reference 2D class averages. For PrP-CAA (Q160X) PHFs and SFs, a new 300-pixel box size without downscaling was used to re-extract with inter-box distance of approximately 15 Å. A box size of 320 pixels was used for GSS (F198S). At this stage, multiple rounds of reference-free 2D classification were carried out to discard suboptimal 2D class averages. The initial 3D reference maps were reconstructed de novo from best 2D class averages of comprising a full helical crossover. The initial round of 3D classification was low-pass-filtered to 60 Å. Several rounds of 3D classification were carried out to remove segments leading to suboptimal 3D class averages with regularization value *T* = 20, total classes *K* = 4. The final selected segments were used for final 3D auto-refinement with optimization of the helical twist and rise to yield 3D map showing clearly visible beta-strand separation and side-chain densities. We used a 10% helical z percentage parameter for post-reconstruction application of helical symmetry. The final reconstructions were sharpened using the standard post-processing procedures in RELION. The RELION helix toolbox was used to improve helical symmetry. Optimized twist and rise parameters for PrP-CAA (Q160X) PHFs, PrP-CAA (Q160X) SFs, and GSS (F198S) PHFs are 4.77 Å and – 1.2°, 4.79 Å and – 1.07°, and 4.85 Å and – 1.23°, respectively. Finally, the overall resolution was calculated from Fourier shell correlations at 0.143 between two independently refined half-maps, employing phase randomization for the convolution effects correction of an optimized, soft-edged solvent mask as implemented in the trueFSC.py program of our *jspr* software [57].

### Model building

The previously deposited structure of PHF (PDB 5O3L) was fitted into the sharpened PrP-CAA (Q160X) and GSS (F198S) PHF density maps using Chimera [47]. The same was done for PrP-CAA (Q160X) SF using the deposited structure of SF (PDB 5O3T). The most central chain of each model (e.g., chain E) was adjusted into the electron density of each map by hand in Coot [9]. Each model was then refined using Rosetta according to a previously established procedure [66].

### Density map and atomic model analysis

For each unique type of filament, the two half-maps were separately sharpened, aligned, scaled, and matched to the same density level for comparison. If the density of a region was not present in one of the half-maps, it was considered noise and not included for final evaluation. Once noise-free density feature was determined in this process, the full dataset maps for PrP-CAA (Q160X) and GSS (F198S) PHFs were globally sharpened with PHENIX [40], aligned, oriented, scaled, and matched to the density of the corresponding AD PHF map (EMD-3741) for comparison. The PrP-CAA (Q160X) SF density map was also processed and compared against the AD SF (EMD-3743) map in a similar manner. The maps were compared at the full map level, dimer level, and the monomer level.

### Data availability

Cryo-EM maps have been deposited in the Electron Microscopy Data Bank (EMDB) under accession numbers EMD-23871 for PrP-CAA (Q160X) PHF, EMD-23890 for PrP-CAA (Q160X) SF, and EMD-23894 for GSS (F198S) PHF case 1. Refined atomic models have been deposited in the Protein Data Bank (PDB) under accession numbers 7MKF for PrP-CAA (Q160X) PHF, 7MKG for PrP-CAA (Q160X) SF, and 7MKH for GSS (F198S) PHF case 1. The mass spectrometry proteomics data generated in this study have been deposited to the ProteomeXchange Consortium [61] via the PRIDE partner repository [46] with the dataset identifier PXD025663. Whole-exome sequencing data have been deposited in the National Institute on Aging Alzheimer’s Disease Data Storage Site (NIAGADS; https://www.niagads.org), under accession number ng00107.

## Results

### Tau aggregates in Prion-Protein Amyloidoses are composed of hyperphosphorylated Tau containing three- and four-repeat Tau isoforms

**Fig. 1:**
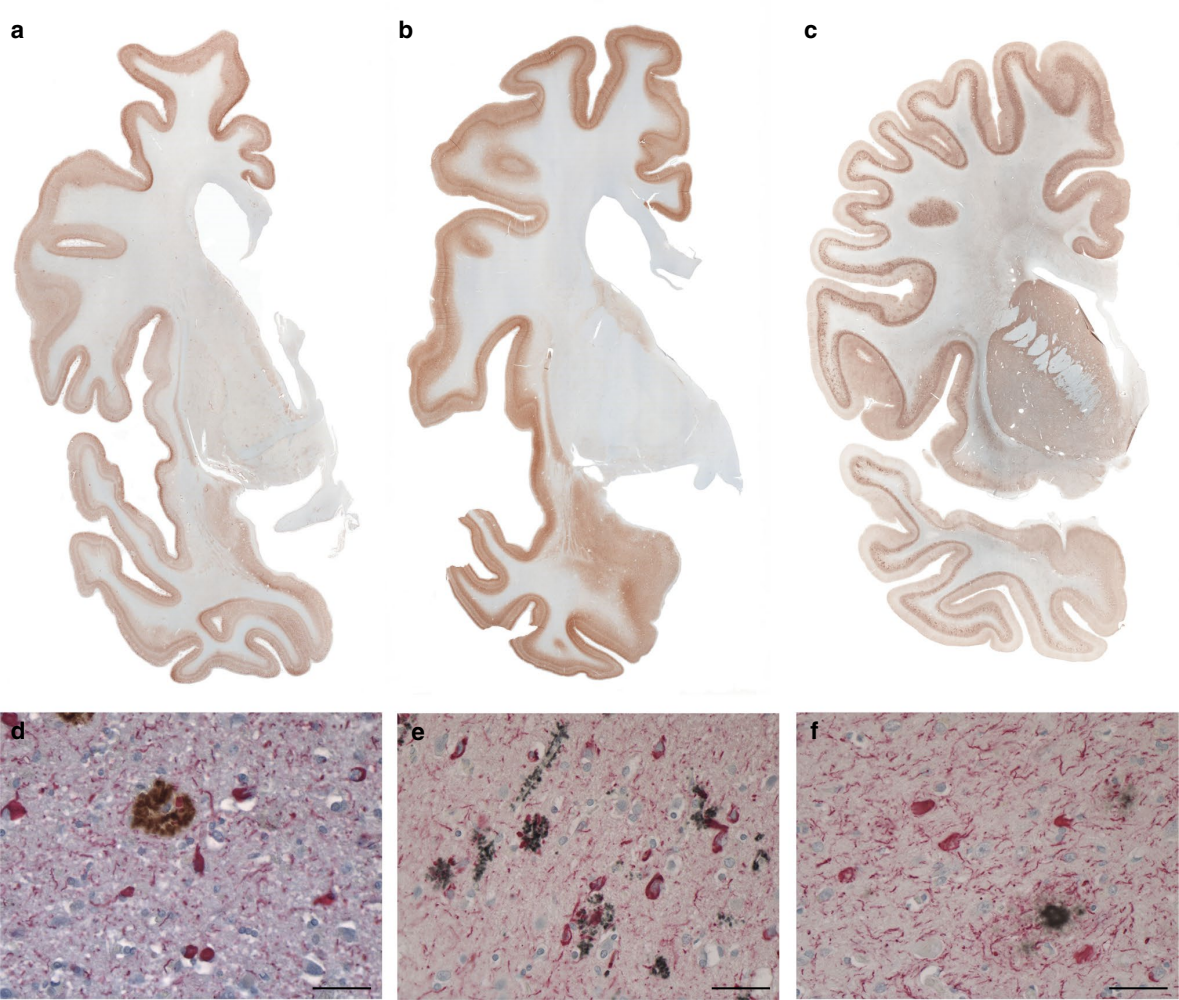
*Immunohistochemistry in PrP-CAA (Q160X) and GSS (F198S) compared to AD.* Hemispheric coronal sections show the distribution of Tau pathology at the level of the cerebral cortex, caudate nucleus and putamen in a case of AD (Case 1, **a**), cerebral cortex, amygdala, and caudate nucleus in PrP-CAA (Q160X) **b**, and cerebral cortex, caudate nucleus, putamen, and claustrum in GSS (F198S) (Case 1, **c**). Double immunohistochemistry of Tau and Aβ in AD (**d**), Tau and PrP in PrP-CAA (Q160X) (**e**), and Tau and PrP in GSS (F198S) (**f**). Nerve cell bodies and NTs are reactive for Tau (red) and are seen in the vicinity of parenchymal Aβ plaques (brown) in AD (**d**), blood vessels with APrP angiopathy (black) in PrP-CAA (Q160X) (**e**), or parenchymal APrP plaques (black) in GSS (F198S) (**f**). **a**–**f**, Anti-Tau antibody AT8; **d** Anti-Aβ antibody NAB 228; **e**, **f** Anti-PrP antibody 95–108. Scale bar, 50 µm

One of the hallmarks of the neuropathology of PrP-CAA (Q160X) and GSS (F198S) is the co-existence of APrP and Tau deposits (Fig. 1, Figure S1). Severe Tau neurofibrillary pathology including NFTs and neuropil threads (NTs) in gray structures of the cerebrum and brainstem coexist in the same anatomical areas with APrP deposition. While APrP pathology is severe in the molecular and granule cell layers of the cerebellar cortex, there is no Tau deposition in any of the cerebellar compartments in either of the two diseases. In PrP-CAA (Q160X), APrP angiopathy coexists with severe limbic and neocortical Tau pathology that, as in GSS (F198S), is also characterized by the presence of numerous NFTs and NTs, that are decorated using antibodies recognizing hyperphosphorylated Tau and 3R- and 4R-Tau (Figure S1). APrP fibrillary deposits are decorated by antibodies recognizing PrP and visualized within vessel walls and intimately adherent to them, often forming rosary-like structures. APrP angiopathy involves the walls of small- and medium-sized parenchymal and leptomeningeal blood vessels. In GSS (F198S), NFTs and NTs are decorated using antibodies recognizing hyperphosphorylated Tau, 3R- and 4R-Tau (Figure S1). Tau deposits are seen in neuronal perikarya, in NTs, and in the processes that surround PrP plaques (Fig. 1, Figure S1). PrP plaques may be diffuse or have cores (unicentric or multicentric plaques) that are predominantly in layers 1, 4, 5, and 6 of frontal, insular, temporal, and parietal cortices. It is noteworthy that Tau pathology is equally severe in PrP-CAA (Q160X) and GSS (F198S), even though the APrP pathology is localized in different compartments: i.e., blood vessels walls in PrP-CAA (Q160X) versus neuropile in GSS (F198S).

**Fig. 2:**
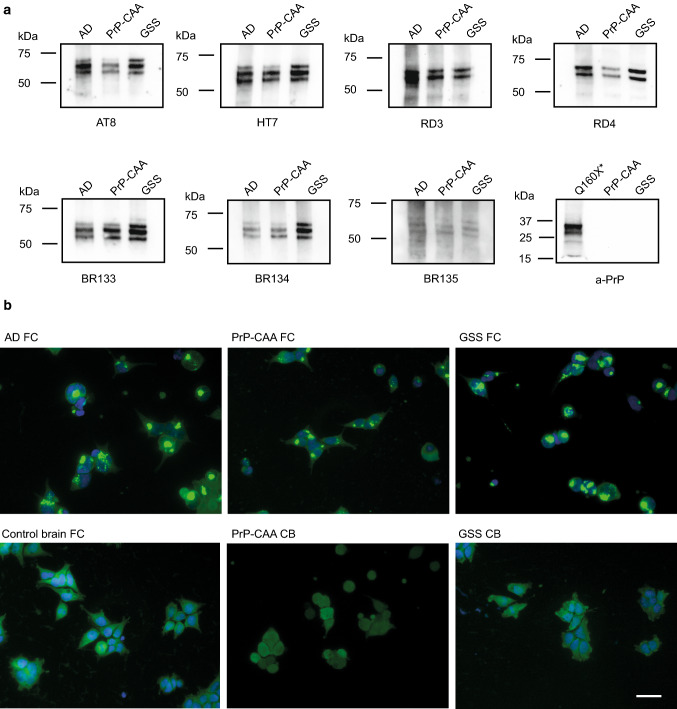
*PrP-CAA (Q160X) and GSS (F198S) Tau are indistinguishable from AD Tau by Western blot or seeding assay.* Western blots of sarkosyl-insoluble Tau fractions from AD (Case 2), PrP-CAA (Q160X), and GSS (F198S) (Case 2) show that Tau aggregates consist of full length, hyperphosphorylated Tau with an identical electrophoretic pattern, consisting of major bands of 60, 64, and 68 kDa. No PrP immunoreactivity is seen in the sarkosyl-insoluble Tau fractions of PrP-CAA (Q160X) and GSS (F198S). Total brain homogenate from PrP-CAA (Q160X*) was used as positive control for PrP (**a**). Tau biosensor cells incubated with the sarkosyl-insoluble fraction obtained from frontal cortex (FC) of AD (Case 2), PrP-CAA (Q160X) and GSS (F198S) (Case 2) show Tau seeding activity, whereas the insoluble fraction of FC from control brain, and cerebellum (CB) from PrP-CAA (Q160X) and GSS do not seed Tau aggregation in vitro (**b**). Scale bar: 20 µm

Western blot analysis of sarkosyl-insoluble fractions shows the presence of Tau bands with a migration pattern indistinguishable from that seen in AD (Fig. 2, Figure S2a). Western blot analysis also shows that APrP does not co-purify with Tau in our Tau preparations (Fig. 2a). The purified sarkosyl-insoluble fraction enriched for Tau aggregates induces Tau aggregation in a biosensor cell system derived by transducing HEK293T cells with 2 separate lentiviral constructs encoding Tau RD P301S-CFP and Tau RD P301S-YFP [18, 33]. Tau isolated from the frontal cortex of PrP-CAA (Q160X) and GSS (F198S) leads to the formation of aggregates similar to those from Tau isolated from AD brains (Fig. 2b). Aggregation is not seen in samples prepared from frontal cortex (FC) from controls nor from the cerebellum (CB) of PrP-CAA (Q160X) and GSS (F198S) patients, where PrP pathology is severe, but Tau aggregates are not detected.

The sarkosyl-insoluble fraction enriched in Tau aggregates from PrP-CAA (Q160X) and GSS (F198S) was incubated in the presence or absence of pronase and evaluated by a dot-blot assay using a variety of antibodies against different epitopes of Tau protein (Figure S2a). We compared the susceptibility of the Tau preparation to pronase digestion in the two diseases to that of Tau fibrils isolated from AD, CTE, CBD, and PiD. Digestion with pronase removes the epitopes recognized by the BR133, HT7 and AT8 antibodies, located in the N-terminal portion of the molecule. It also removes the epitope recognized by the BR134 antibody, located in the C-terminus of Tau. Both N- and C-terminal portions of Tau are also referred to as the “fuzzy coat” [68]. Interestingly, the epitope recognized by the BR135 antibody is partially resistant to pronase digestion, suggesting that this sequence is part of the filament core as in AD, CTE, CBD, and PiD (Figure S2b) [10–12, 16, 64].

Analysis of dispersed preparations of Tau filaments by transmission electron microscopy (TEM) suggests that Tau aggregates from GSS (F198S) may be composed predominantly of paired helical filaments (PHFs) that appear to be identical to those seen in AD (Figure S2c). In PrP-CAA (Q160X), we estimate a ratio of ~ 4:1 PHFs to straight filament (SFs), while SFs could not be detected in the three different GSS (F198S) cases analyzed, suggesting that PHFs may be the predominant (if not the only) type of filaments in GSS (F198S). Using antibodies against different epitopes of Tau protein (Figure S2a) in immuno-EM, we observe that Tau filaments in PrP-CAA (Q160X) and GSS (F198S) are composed of full-length, hyperphosphorylated Tau. Immuno-EM analysis of Tau fibrils after pronase digestion shows that the cores of Tau filaments in PrP-CAA (Q160X) and GSS (F198S) share epitopes located in the R3 and R4 repeat domains. Treatment with pronase removes the fuzzy coat, composed of N- and C-terminal sequences, abolishing the positive labeling observed before pronase treatment. The epitope recognized by antibody BR135, which labels Tau on Western blots of dispersed filaments and in dot-blot analyses, is located in the core of Tau filaments, and is not accessible to the antibody before or after pronase treatment by immuno-EM (Figure S2c). These findings are consistent with the presence of the same Tau sequences in the core of Tau filaments in PrP-CAA (Q160X), GSS (F198S), and AD [11, 16].

### Cryo-EM of Tau filaments in PrP-CAA (Q160X) and GSS (F198S)

**Fig. 3:**
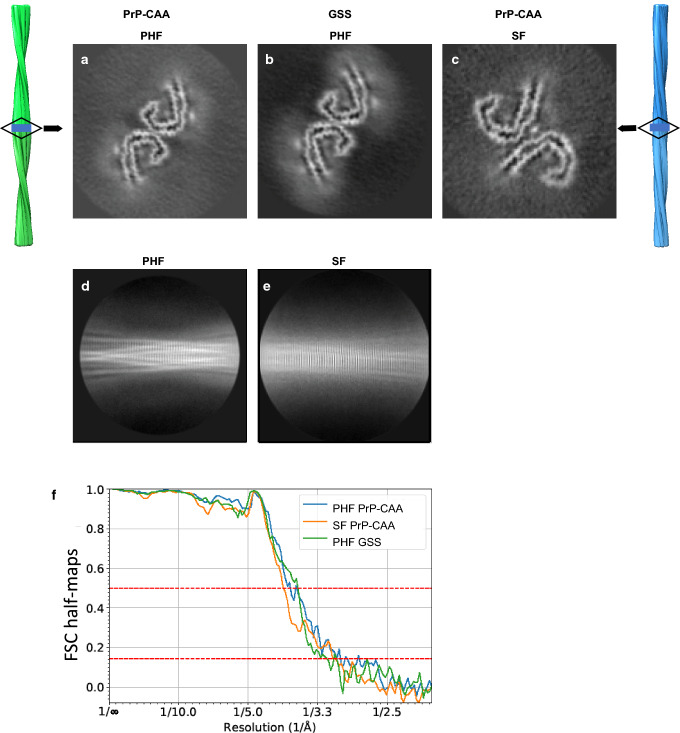
*Cryo-EM reconstructions of PHF and SF from PrP-CAA (Q160X) and PHF from GSS (F198S).* The structures show identical pairs of C-shaped protofilaments and the same inter-protofilament packing between PHFs (**a**, **b**) but different packing for SFs (**c**). The rectangular boxes in the vertical, surface view of the helical reconstructions of PHFs (left, green), and SFs (right, blue) show the location of the cross-sectional densities (**a**–**c**). 2D class averages of PHF (**d**) and SF (**e**) of PrP-CAA (Q160X). Fourier Shell Correlation (FSC) curves for independently refined half-maps of PrP-CAA (Q160X) PHFs and SFs, and GSS (F198S) PHFs (**f**)

We determine the structure of Tau filaments at high resolution by cryo-EM imaging and 3D reconstruction. We observe two types of Tau filaments within the sarkosyl-insoluble fraction of PrP-CAA (Q160X), similar to PHFs and SFs of AD (Fig. 3a,c). PHFs were ~ 70% and SFs were ~ 30% of the filament population, both composed of two protofilaments with C-shaped subunits (Figure S3) [16]. Two-dimensional classification readily separated PHF and SF for further processing (Fig. 3d,e). We determine the structure of PHFs and SFs in PrP-CAA (Q160X) using helical reconstruction in RELION3.1 to 3.0 Å resolution (Fig. 3a, c, f) (Table S1). The crossover distance for both types of filaments is approximately between 700 and 850 Å with a width of about 40–300 Å. In GSS (F198S), the protofilament organization of PHFs is identical to that of PrP-CAA protofilaments; however, protofilaments of SF were not visualized. Using helical reconstruction in RELION3.1, we determine a 3.2 Å resolution map of the ordered core of PHFs from GSS (F198S) (Fig. 3b, f), with the same crossover distance and width of PHFs from PrP-CAA (Q160X), and the same monomeric fold as the AD fold, i.e., the C-shaped unit (Figure S3).

**Fig. 4:**
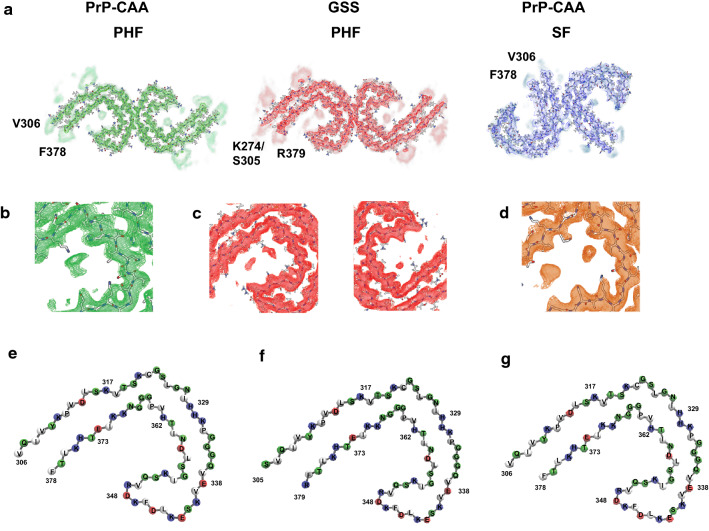
*Cryo-EM densities and atomic models of PHFs and SFs.* Sharpened, high-resolution maps are shown in green (PHF, PrP-CAA (Q160X)), red (PHF, GSS (F198S)), and blue (SF, PrP-CAA (Q160X)). The blurred map regions represent extra densities low-pass-filtered to 5 Å (**a**). PrP-CAA (Q160X) PHF extra density (**b**) and GSS (F198S) extra density (**c**) around the core have different locations and orientations to that of the extra density of AD PHF (**d**). Schematic view of the PrP-CAA (Q160X) (**e**) and GSS (F198S) (**f**) PHF protofilament cores compared to AD (**g**) PHF (PDB: 5o3l) showing the similarities in the folds. The colors represent the polarity of the amino acids (red: negatively charged, blue: positively charged, white: non-polar)

The cores are composed of eight β-strands (Figure S3, S4a) adopting a C-shaped architecture that is perpendicular to the axial direction of the filament encompassing residues V_306_ to F_378_ of Tau in PrP-CAA (Q160X), and K_274_ to R_379_ of 3R Tau and S_305_ to R_379_ of 4R Tau in GSS (F198S) (Figure S3). The cores include residues located in the R3 and R4 repeat domains, with additional densities extending from the N- and C-terminal regions of the core (Figure S4a). The polar amino acids have side chains mostly facing the solvent (outside the protofilament core) and the non-polar amino acids like valine and isoleucine are facing inwards in the protofilament core forming hydrophobic patches (Fig. 4). PHFs and SFs in PrP-CAA (Q160X) and PHFs in GSS (F198S) have 8 β-strands that pack together to form the fold (Figure S3i-k). The β1 and β2 strands run anti-parallel to β8 (in a cross-β pattern), β3 runs anti-parallel to β7, and β4, β5 and β6 are arranged in a triangular fashion. In between the β-strands, glycine residues forming β-turns and proline residues breaking the β-strands reduce the stress of having 8 β-strands in one protofilament core (Fig. 4). Ordered in-register H-bonding between the residues in the β-strands further reduce this stress. The residues in β1 are mostly hydrophobic, packing with the last few residues of β8 strand (L_376_ and F_378_). Contrary to that, the polar residues in the β2 strand are facing inwards to interact with the polar residues of the β8 strand (Figure S3, Fig. 4). A β-turn consisting of G_323_ and G_326_ separates the β2 and β3 strands and provides a 90degree turn to the core. The β3 strand is followed by the _332_PGGG motif that forms an interface with the other protofilament forming a PHF (Figure S4b) and provides another turn in the core structure. V_337_ and V_339_ from the β4 strand interact with I_354_ of the β6 strand and L_357_ of β7 strand to form another hydrophobic patch. The β4–β6 strands form a β-helix-like configuration where the three strands form a triangle-like structure (Fig. 4, Figure S3). The inside of the β-helix is filled with hydrophobic amino acid side chains (V_339_, L_344_, F_346_, V_350_, and I_354_). The side chains of the polar amino acids are facing outwards and they are mostly alternatively charged. E_342_–K_343_ and K_347_–R_349_ residues form the two corners of the triangle. G_355_ provides the turn between β6 and β7 strands. The β7 and β8 strands are interspersed by the _364_PGGG motif that provides the 90 degree turn between them. PHFs and SFs differ in how the two C-shaped protofilaments interact with each other (Fig. 4, Figure S4b). In PHFs, the two protofilaments are related by a 2_1_ screw symmetry with only a small shift (~ 2.4 Å) along the filament axis, which results in the two C-shaped subunits appearing to be inverted and symmetrically interacting with each other (Fig. 4). The interface between protofilaments (Figure S4b) is formed by the anti-parallel stacking of residues P_332_ to Q_336_, with the G residues 333 to 335 forming H-bondings, and two additional H-bonds between Q_336_ and K_331_ from the opposite protofilament. The protofilament surface of PrP-CAA (Q160X) and GSS (F198S) PHFs is similar to the filament surface of PHFs in AD (Figure S4B). The two protofilaments in SF structures are asymmetrically arranged. In SFs, the sidechains of residues P_312_–K_321_ of one protofilament are in close proximity with the sidechains of the residues K_317_–S_324_ of the other protofilament, while the surface between the two protofilaments is not stabilized by H-bonds like in PHFs.

### Additional densities exist around the protofilament core

We observe additional densities around the protofilament cores (Figs. 3, 4). As illustrated in Fig. 4 by the low-pass filtered density map, both N- and C-termini of the cores have disordered densities. In the cores of PHFs in PrP-CAA (Q160X) and GSS (F198S), the sidechains of K_317_, T_319_ and K_321_ interact with an additional density outside the core. Similarly, this strong additional density is seen in the SFs of PrP-CAA (Q160X) at the protofilament interface. Fitzpatrick et al. [5] hypothesized these additional densities to be the _7_EFE_9_ motif at the N-terminus of the Tau protein and proposed that the formation of salt bridges stabilized the interaction between two SF protofilaments. To further investigate the extra densities seen near residues within the core of Tau PHFs and SFs, we perform mass spectrometry (MS) on Tau filaments from AD, PrP-CAA (Q160X) and GSS (F198S). We achieve 100% coverage of the core region of filaments from all 3 diseases. We determine Tau filaments in each disease to be highly post-translationally modified, especially in respect to phosphorylation, acetylation, ubiquitination, and deamidation (Table S3). Specifically, we find peptides in which K_317_ is ubiquitinated in Tau filaments from all 3 diseases, which may correlate to the large extra density near this residue in both PHFs and SFs, therefore suggesting that ubiquitination of K_317_ may also contribute to SF stabilization. A density present near residues I_371_ and T_373_ in PHFs and SFs in AD is not present in Tau filaments from PrP-CAA (Q160X) (Fig. 4) nor do we find any PTMs on these residues via MS (Table S3). In addition, a density that interacts with the H_362_ residue side-chain in Tau filaments in AD is seen to interact with the side-chain of K_369_ in Tau filaments from PrP-CAA (Q160X) and GSS (F198S) (Fig. 4). In fact, our MS results determine that K_369_ is acetylated in both AD and PrP-CAA (Q160X), whereas no PTMs are observed on H_362_, suggesting that this extra density may be interacting with K_369_ as opposed to H_362_. Interestingly, Tau in GSS (F198S) seems to be less phosphorylated at serine residues (S_320_, S_324_ and S_356_) located in the R3 and R4 region than in Tau filaments from AD and PrP-CAA (Q160X) (Table S3).

## Discussion

PrP-CAA (Q160X) and GSS (F198S) are two Prion-Protein Amyloidoses that have distinct clinical and neuropathologic phenotypes. They differ significantly from Creutzfeldt–Jakob disease (CJD), the most common form of prion disease [22]. PrP-CAA (Q160X) and GSS (F198S) are caused by mutations in the *PRNP* gene that lead to APrP formation and deposition in the vascular compartment in PrP-CAA (Q160X) and in the brain parenchyma in GSS (F198S) [21, 23, 37]. Interestingly, both conditions are neuropathologically characterized by the presence of Tau aggregates. Western blot of Tau aggregates from PrP-CAA (Q160X), GSS (F198S), and AD show identical pattern of migration and immunoreactivity using antibodies against 3R- and 4R-au, strongly suggesting that Tau aggregates in PrP-CAA (Q160X) and GSS (F198S) are composed by 3R- and 4R-au isoforms. By TEM of negative stained preparations, Tau aggregates in PrP-CAA (Q160X) are found to be composed of PHFs and SFs, while in GSS (F198S) Tau aggregates are found to be predominantly, if not entirely, composed of PHFs. Analysis by immuno-EM coupled to pronase treatment suggests that the core of the Tau filaments in PrP-CAA (Q160X) and GSS (F198S) contains the R3 and R4 repeats, as in AD. By cryo-EM, PHFs and SFs are found to be made of two protofilaments with a common cross-β/β-helix C-shaped architecture, as in AD [11, 16]. The core encompasses residues V_306_ to F_378_ of Tau in PrP-CAA (Q160X), and K_274_ to R_379_ of 3R Tau and S_305_ to R_379_ of 4R-Tau in GSS (F198S), with disordered densities sensitive to pronase degradation extending from the N- and C- termini. This is similar to the recently reported high-resolution cryo-EM structures of PHFs and SFs from AD, with R_379_ and E_380_ from the sequence after R4 at the C-terminus, and G_304_ and S_305_ from R2 in 4R Tau, and G_273_ and K_274_ from R1 in 3R-Tau at the N-terminus [11]. Additional densities are identified near the core of Tau filaments from PrP-CAA (Q160X) and GSS (F198S), specifically at the sidechains of K_317_, T_319_, and K_321_. By mass spectrometry analysis, we establish the presence of extensive PTMs in Tau aggregates from PrP-CAA (Q160X) and GSS (F198S). We observe K_317_ to be ubiquitinated in AD, PrP-CAA (Q160X) and GSS (F198S), suggesting that the extra densities observed by cryo-EM near this residue in PrP-CAA (Q160X) and GSS (F198S) correspond to ubiquitination at this lysine. We also observe PTMs that have been reported to be important in Tau fibrillization and propagation in AD [27, 28], for example, phosphorylation at T_231_, thought to be central to the initial steps of Tau detachment from microtubules and subsequent aggregation [1, 2], is found in our cases of AD, PrP-CAA (Q160X) and GSS (F198S). We also observe PTMs within the two hexapeptide motifs, _275_VQIINK_280_ and _306_VQIVYK_310_, which are known to be the minimum required motifs for Tau protein aggregation [39]. By MS, we observe that Tau from PrP-CAA (Q160X) is phosphorylated at S_199_ and S_202_, while Tau from GSS (F198S) is phosphorylated at S_202_; however, Tau deposits were immunoreactive for the AT8 phosphoepitope (phosphorylation at residues S_202_ and T_205_ but also other epitopes, including S_199_ and S_208_) [41] by immunohistochemistry, immunoblotting, and immuno-EM methods. PHF-1 also decorates Tau aggregates in PrP-CAA (Q160X) and GSS (F198S) (not shown), and by MS, we are able to detect the phosphorylated residues S_396_ and S_404_, which constitute the epitope recognized by antibody PHF-1 (directed against the doubly phosphorylated epitope S_396_ and S_404_) [29, 32]. The MS data also show deamidation of N_279_, which has been suggested to contribute to the AD folds as opposed to the folds seen in CBD [3, 7]. However, we observe deamidation at this residue only in AD and PrP-CAA (Q160X), but not in GSS (F198S) tryptic peptides, suggesting that this PTM may contribute to PHF over SF formation, and offer explanation as to the lack of SFs in GSS (F198S). In addition, we observe phosphorylation at T_175_ and S_237_ in all three diseases. It has recently been reported that singly and doubly ubiquitinated peptides at residues K_311_ and K_317_ and phosphorylation at T_217_ and S_262_ may differentiate between AD and control groups [67]. We find that all these residues are post-translationally modified in PrP-CAA (Q160X) and GSS (F198S). We observe peptides containing K_311_ to be either ubiquitinated or acetylated, while K_317_ is always found ubiquitinated. Although we do not find changes in K_280_, PTMs in K_280_ and K_311_ have been shown to regulate Tau fibrillization in vitro [62]. It has been previously suggested that extra densities near K_317_ and K_321_ of AD and CTE Tau may be formed by the _7_EFE_9_ motif of Tau, which may stabilize the protofilament interface of SFs [12, 16]. We identify ubiquitination of K_317_ in PrP-CAA (Q160X) and GSS (F198S) Tau, suggesting ubiquitin at this site as another possible explanation for the extra density seen via cryo-EM. Additional reports have suggested that ubiquitination of K_311_ and K_317_ of the core of Tau filaments would lead to SF formation, rather than PHF [7]; however, our data show that despite ubiquitination at K_311_ and K_317_ of tryptic peptides from GSS (F198S), no SFs are found within GSS (F198S) Tau filaments. In addition, we do not find peptides phosphorylated at S_320_, S_324_ and S_356_ in GSS (F198S), which are located in the R3 and R4 region and are phosphorylated in AD and PrP-CAA (Q160X). Our data suggest that these specific PTMs may not be critical for SF formation, and that PTMs may not fully mediate the structural diversity seen between Tau filaments from different diseases. Phosphorylation at T_175_, S_237_, and ubiquitination at K_281_, have been associated with symptomatic AD at Braak VI stage [5, 67]

**Fig. 5:**
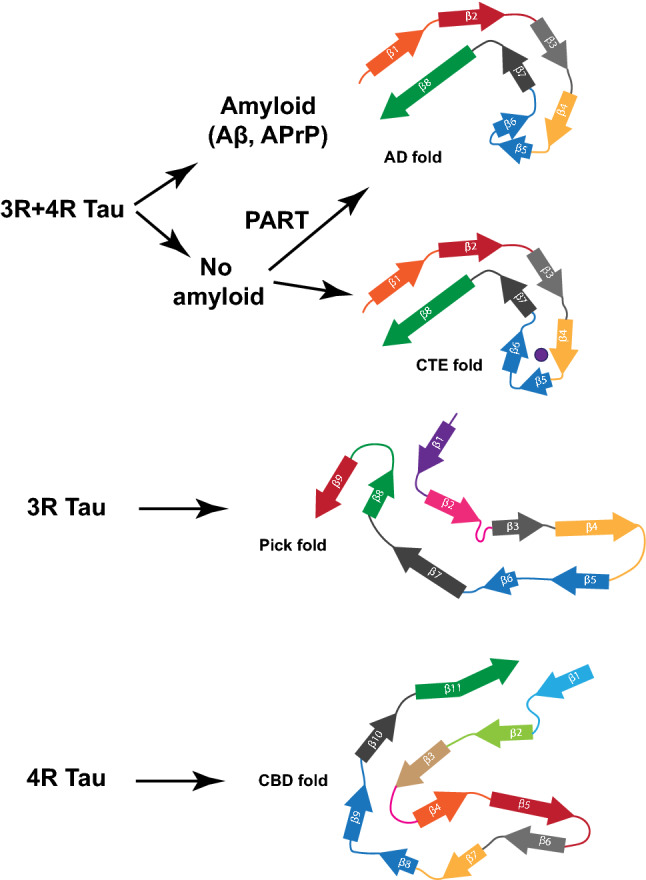
*Different folds of Tau that have been identified to-date*. Two different Tau folds are associated with 3R and 4R-Tau and make the Tau aggregates in AD, PrP-CAA (Q160X), GSS (F198S), PART, and CTE. A Tau fold associated with 3R-Tau makes the inclusions of Pick disease and a Tau fold associated with 4R-Tau makes the aggregates in CBD. Among the four folds, one known as the Alzheimer Tau fold can occur in the presence or in the absence of an extracellular amyloid deposition. Analysis of additional diseases characterized by 3R-, 4R-, and 3R and 4R-Tau will determine whether this is the complete Tau fold landscape or if additional folds may be found associated with different diseases

Amyloid and Tau aggregates coexist in AD and in other diseases in addition to the group of the PrP Amyloidoses [22], two of which are reported here. In fact, in other hereditary cerebral amyloid diseases such as Familial British dementia (FBD) [34, 59] and Familial Danish dementia (FDD) [35, 60], a severe neurofibrillary Tau pathology occurs. Our study shows for the first time that Tau fibrils deposited in the brain of individuals with a brain amyloidosis other than AD are biochemically, antigenically, and structurally identical. Moreover, a recent study shows that Tau fibrils isolated from the brain of individuals with FBD and FDD are also structurally identical to those in AD [56]. The co-existence of Tau aggregates with different types of amyloids suggests a common mechanism through which amyloids, whether Aβ in AD, APrP in Prion diseases, ABri in FBD or ADan in FDD, trigger aggregation of Tau, resulting in Tau filaments with identical structure at their core (Fig. 5). Furthermore, Tau from the brains of patients with AD, GSS (F198S), and PrP-CAA (Q160X) have similar seeding activities in vitro, as has been also seen for brain homogenates from AD and PART [38]. For AD, it has been proposed that Aβ provides a crucial element toward Tau aggregation [4, 30]. This hypothesis has been supported by genetic forms of AD due to mutations in the *AβPP*, *PSEN1*, and *PSEN2* genes that consistently alter the metabolism of Aβ, with a consequent Tau hyperphosphorylation and formation of Tau aggregates in vitro and in vivo [15, 19, 28]. Altered Tau metabolism in association with APrP has also been observed in in vitro studies [42] and in vivo in mouse models [48, 50]. By determining the structure of the core of Tau filaments from diseases caused by two distinct *PRNP* mutations, F198S and Q160X, to be identical to the core of Tau filaments from AD, we uncover potential links between amyloid proteins and the resulting Tau aggregation. Structural data are urgently needed for the identification of specific ligands for in vivo imaging of Tau aggregates in a wide range of neurodegenerative diseases.

## Supplementary Information

Below is the link to the electronic supplementary material.

Supplementary file1 (PDF 161675 KB)
