# Supplementary material for: Structure of Tau filaments in Prion protein amyloidoses
Source: Acta Neuropathol. Author manuscript; Available in PMC 2021 Aug 1. (PMC8270882; doi:10.1007/s00401-021-02336-w)
Supplement: Hallinan SM [file NIHMS1721892-supplement-Hallinan_SM.pdf]

Structure of Tau filaments in Prion protein amyloidoses

Grace I. Hallinan[1,5], Md Rejaul Hoq[2,5], Manali Ghosh[2], Frank S. Vago[2], Anllely Fernandez[1], Holly J. Garringer[1], Ruben Vidal[1,3,4,6], Wen Jiang[2,4,6], Bernardino Ghetti[1,3,4,6]

[1]Department of Pathology and Laboratory Medicine, Indiana University School of Medicine, Indianapolis, IN 46202, USA
[2]Department of Biological Sciences, Markey Center for Structural Biology, Purdue University, West Lafayette, IN 47906, USA
[3]Stark Neurosciences Research Institute, Indiana University School of Medicine, Indianapolis, IN 46202, USA
[4]These authors jointly supervised this work: Ruben Vidal, Wen Jiang, Bernardino Ghetti
[5]Grace I. Hallinan and Rejaul Hoq contributed equally to this work.
[6]Correspondence: Dr. Ruben Vidal, Dept. of Pathology and Laboratory Medicine, Indiana University School of Medicine, 635 Barnhill Dr., MSB A136, Indianapolis, IN 46202; Tel.: 317-274-1729; E-mail: rvidal@iupui.edu; Dr. Wen Jiang, Department of Biological Sciences, Markey Center for Structural Biology, Purdue University, West Lafayette, IN, 47906, USA, E-mail: jiang12@purdue.edu; Dr. Bernardino Ghetti, Dept. of Pathology and Laboratory Medicine, Indiana University School of Medicine, 635 Barnhill Dr., MSB A138, Indianapolis, IN 46202; Tel.: 317-274-7818; E-mail: bghetti@iupui.edu

Supplementary Materials and Methods

Human Tissues. Human brain samples and clinical records were collected before the research was proposed to the institutional review board to determine whether the research is exempt (Category 4) under an IRB-approved autopsy protocol. Subjects cannot be identified, directly or through identifiers linked to the subjects. All work using human materials or samples derived from humans was handled at BSL2. Both sites, IUSM and Purdue University are approved to work at BSL2.

Experimental model and subject details.

Clinical history and neuropathology.

Clinical history and pathology of GSS (F198S) case 1. The patient was a 49-year-old woman with family history of neurological disease. Upon neurological examination, at age 42, she presented with dystaxia, mild rigidity and mild memory loss. Subsequently, she had a six-year history of progressive ataxia bradykinesia, dysarthria, memory deficits. Upon neurological examination, at age 49, the patient exhibited ataxia, marked dysarthria, generalized bradykinesia, rigidity, and masked facies. Ocular motility was abnormal with limitation of upward gaze and hypometria of horizontal and vertical saccades. Formal neuropsychological testing showed mild depression, IQ in the defective range, acquired cognitive impairment, impaired memory processes, deficit in manual speed and dexterity. Upon progression of the disease, ataxia and Parkinsonism became severe. She sustained fractures of the right and left hip. Terminally she refused to eat and had decubitus. At autopsy, the weight of the fresh whole brain was 1,130 g. The brain was hemisected along the midline. The left hemibrain was almost entirely

frozen and stored at -80°C, whereas the right hemibrain was fixed in formalin. On macroscopical examination, the frontal and parietal lobes and the cerebellum revealed a moderate atrophy. The substantia nigra had a decreased pigmentation. On coronal sections the lateral ventricles appeared mildly enlarged and the head of the caudate nucleus mildly atrophic. Histological studies revealed moderate to severe neuronal loss and gliosis in the neocortex, basal ganglia, globus pallidus, substantia innominata, amygdala, thalamus, hippocampus, subiculum, entorhinal cortex, cerebellar cortex, midbrain, pons, and medulla. Axonal and myelin loss were observed in the hemispheric white matter. Immunohistochemical studies revealed numerous unicentric and multicentric amyloid plaques throughout the cerebral and cerebellar cortex, subcortical nuclei and brainstem. The amyloid plaques were labelled with antibodies to PrP. In the same areas where PrP deposits were present, Tau immunohistochemistry revealed numerous NFTs, Tau immunopositive neurons and NTs. Tau pathology coexisted with PrP amyloid with exception of the cerebellum, where Tau pathology was not detected. Aβ immunopositive plaques were absent throughout the CNS. The neuropathologic diagnosis was Gerstmann-Sträussler-Scheinker disease. Whole-exome sequencing did not detect mutations known to cause AD, Parkinson disease, frontotemporal dementias or amyotrophic lateral sclerosis. GSS (F198S) Case 1 had a single nucleotide (T to C) substitution in codon 198 of one allele of the *PRNP* gene. This change results in a serine for phenylalanine amino acid change (F198S). For this patient, the first base of *PRNP* codon 129 was homozygous guanine (G), coding for valine (GTG). The APOE genotype was ε2/ε3.

Clinical history and pathology of GSS (F198S) case 2. The patient was a 53-year-old man with family history of neurological disease. At age 51, on his first visit, the patient had a one-year history of gradually progressive memory and language disorder and a six-month history of difficulties with judgment and reasoning, accompanied by changes in personality characterized by irritability, withdrawal, sadness, socially inappropriate behavior, and agitation. On neurological examination, there was slight tremor, bradykinesia, and ataxic gait. The global Clinical Dementia Rating (CDR) was 0.5, indicating mild global impairment, with mild impairment (a 0.5 rating) in the memory, orientation, judgement and problem-solving, community affairs, and home and hobbies domains, and moderate impairment (a 1.0 rating) in the behavior, comportment, and personality domains of the supplemental CDR. The Neuropsychiatric Inventory indicated the presence of agitation, depression, anxiety, disinhibition, irritability, nighttime behaviors, and problems with appetite with severity scores predominantly in the mild range. The FAS indicated mild impairment in daily functioning. The neuropsychological battery revealed mild impairments in efficiency of new learning, verbal fluency, response inhibition, complex sequential tracking, and manual motor speed. The Mini-Mental State Examination score was 26/30. On follow up neurological examination, there was evidence of Parkinsonism and gait disturbances. The global CDR had progressed to 1.0, indicating moderate impairment, with mild impairment (a 0.5 rating) in memory and orientation domains, mild to moderate impairment (a 1.0 rating) in judgement and problem-solving, community affairs, and personal care domains, and severe impairment (a 3.0 rating) in the home and hobbies domain. The NPI now included delusions in addition to the problems noted on the previous visit, with

most symptoms rated as moderate in severity. The FAS total score had progressed to 13, with need for assistance in shopping, simple meal preparation and cooking, and traveling. The second neuropsychological examination revealed mild to moderate declines in new learning, memory, executive cognitive function, psychomotor speed, and manual motor skills. The MoCA score was 18/30 and GDS was within normal limits. The consensus diagnosis was dementia due to GSS. On macroscopical examination, the frontal and parietal lobes and the cerebellum revealed a moderate atrophy. The brain was hemisected along the midline. The left hemibrain was frozen and stored at -80°C, whereas the right hemibrain was fixed in formalin. At autopsy, the weight of the fresh whole brain was 1,452 g. Neuronal loss and gliosis, moderate to severe, was observed in the cerebral cortex, basal ganglia, globus pallidus, substantia innominata, amygdala, thalamus, hippocampus, subiculum, entorhinal cortex, cerebellar cortex, midbrain, and pons. Severe axonal and myelin loss was observed in hemispheric and cerebellar white matter. Immunohistochemical studies revealed numerous unicentric and multicentric PrP amyloid plaques in the form of unicentric and multicentric plaques, in the neocortex, basal ganglia, globus pallidus, substantia innominata, amygdala, thalamus, hippocampus, subiculum, entorhinal cortex, cerebellar cortex, dentate nucleus, midbrain, pons, and medulla. NFTs, Tau immunopositive neurons and NTs were observed in the cerebral cortex, basal ganglia, amygdala, subiculum, and entorhinal cortex. Severe arteriolosclerosis was observed in hemispheric white matter and basal ganglia. The neuropathologic diagnosis was Gerstmann-Sträussler-Scheinker disease. Whole-exome sequencing did not detect mutations known to cause AD, Parkinson disease, frontotemporal dementias or amyotrophic lateral sclerosis. GSS

(F198S) Case 2 had a single nucleotide (T to C) substitution in codon 198 of one allele of the *PRNP* gene. This change results in a serine for phenylalanine amino acid change (F198S). For this patient, the first base of *PRNP* codon 129 was homozygous guanine (G), coding for valine (GTG). The APOE genotype was ε2/ε3.

Clinical history and pathology of GSS (F198S) case 3. The patient was a 71- year-old man with family history of neurological disease. Upon neurological examination, at age 60, he presented with mild dystaxia and anxiety. Formal neuropsychological testing showed average IQ, but deficient sustained concentration. On his neuropsychological testing, he had an average level of general intellectual functioning, tests of sustained concentration were deficient. He was impaired in tests with immediate delayed recall of paragraph length stories, immediate reproduction of geometric figures and conceptual reasoning. He had bilateral deficits and manual speed and dexterity. These test deficits were thought to represent impairments in sustained attention, verbal memory, abstract, problem solving and manual motor performance. He was next seen at age 61. He had been having particular difficulties with balance and coordination and these had particularly impaired his gait. He was having increased problems with writing, using utensils at the table and had increasing memory problems. On his examination, there were mild difficulties with memory, moderate bradykinesia and rigidity in all his extremities. He was followed intermittently. At age 66, he had progressed rather dramatically and had to have a feeding tube placed because he was having difficulty eating food and was choking. He was mute, communicating with facial twitches, open and closing of his eyes, not able to use his arms and legs in a useful manner, was

getting contraction of his extremities, was very stiff. He had dementia; however, it was difficult to assess his cognitive condition due to the very severe extrapyramidal syndrome. He was seen at age 67, when he was again bound with G tube and no real possibility of useful communication. He appeared disinterested, but he would respond emotionally to speech with smiles or crying making dysarthric noise, but really nothing understandable. He continued to be seen every 12 to 18 months. The neurological conditions and the dementia progressed, and he died 11 years following the onset of his symptoms. At autopsy, the weight of the fresh whole brain was 1,020 g. The brain was hemisected along the midline. The left hemibrain was frozen and stored at -80°C, whereas the right hemibrain was fixed in formalin. On macroscopical examination, the frontal and parietal lobes and the cerebellum revealed a moderate atrophy. The substantia nigra was severely atrophic and depigmented. On coronal sections the lateral ventricles appeared enlarged and the head and body of the caudate nucleus severely atrophic. Histological studies revealed neuronal loss and gliosis in the neocortex, basal ganglia, globus pallidus, substantia innominata, amygdala, thalamus, hippocampus, subiculum, entorhinal cortex, cerebellar cortex, midbrain, pons, and medulla. Axonal and myelin loss were observed in the hemispheric white matter. Immunohistochemical studies revealed numerous unicentric and multicentric PrP amyloid plaques throughout the cerebral and cerebellar cortex, subcortical nuclei and brainstem. In the same areas where PrP deposits were present, Tau immunohistochemistry revealed numerous NFTs, Tau immunopositive neurons and NTs. Tau pathology coexisted with PrP amyloid with exception of the cerebellum, where tau pathology was not detected. Immunohistochemistry for alpha-synuclein revealed

numerous Lewy body and neurites throughout the cerebral cortex, subcortical nuclei and brainstem. Aβ immunopositive plaques were absent throughout the CNS. The neuropathologic diagnosis was Gerstmann-Sträussler-Scheinker disease. Whole-exome sequencing did not detect mutations known to cause AD, Parkinson disease, frontotemporal dementias or amyotrophic lateral sclerosis. GSS (F198S) Case 3 had a single nucleotide (T to C) substitution in codon 198 of one allele of the *PRNP* gene. This change results in a serine for phenylalanine amino acid change (F198S). For this patient, the first base of *PRNP* codon 129 was heterozygous guanine/adenine (G/A), coding for valine/methionine (GTG/ATG). The APOE genotype was ε3/ε3.

Clinical history and pathology of PrP-CAA (Q160X). A 33-year-old male, who presented with a frontal syndrome, gastrointestinal symptoms, and peripheral neuropathy at age 27. He had a slowly progressive dementia. Behavior, memory, executive function, speech/language, and motor function were severely affected. He died at age 33. The clinical syndrome of the patient has been described [17]. The weight of the fresh brain was 1,055 g. Atrophy was most severe at the level of the frontal and temporal lobes. The fresh brain was hemisected along the mid-sagittal plane. The right cerebral hemisphere, right cerebellar hemisphere and the right half of brain stem were frozen and stored at -80°C. The left hemibrain was fixed for neuropathologic studies. The head of the caudate nucleus, the putamen and the amygdala were atrophic. The white matter of the centrum semiovale was reduced in bulk. The substantia nigra was moderately depigmented. Histologic and immunohistochemical studies revealed moderate-to-severe, nerve cell loss and gliosis throughout the cerebral cortex,

claustrum, substantia innominate, amygdala, hippocampus, putamen, caudate nucleus, globus pallidus, thalamus, substantia nigra, cerebellum, and spinal cord. Axonal and myelin loss were most evident in the temporal lobe, but were also present in the frontal, parietal and occipital lobes, cerebellum and spinal cord. PrP deposits were observed in association with blood vessel wall throughout the cerebral cortex, amygdala, hippocampus, claustrum, putamen, caudate nucleus, thalamus, substantia innominata, cerebellum, and spinal cord. Severe Tau-immunoreactive neurons and NTs were observed in the same areas affected by PrP pathology with exception of the cerebellum, where tau pathology was not detected. Severe NFT pathology was observed in the temporal neocortex, hippocampus, entorhinal cortex, amygdala. Moderate NFT pathology was observed in the frontal neocortex, insular cortex, and cingulate cortex, while mild NFT pathology was observed in the parietal neocortex, with rare pathology seen in the basal ganglia and thalamus. Mild arteriolosclerosis was observed in the basal ganglia, frontal lobe, temporal lobe. Neuropathologic diagnosis was Prion Protein Cerebral Amyloid Angiopathy. Whole-exome sequencing did not detect mutations known to cause AD, Parkinson disease, frontotemporal dementias or amyotrophic lateral sclerosis. PrP-CAA (Q160X) Case 1 had a single nucleotide (C to T) substitution in codon 160 of one allele of the *PRNP* gene. This change results in a Gln160-to-ter (Q160Ter) substitution. For this patient, the first base of *PRNP* codon 129 was homozygous adenine (A), coding for methionine (ATG). The APOE genotype was ε3/ε3.

Clinical history and pathology of Alzheimer disease Case 1. The patient was a 49-year-old woman with family history of dominantly inherited AD in several family members

[13]. The patient was a school teacher and had been privately followed since age 40, when a mutation causing AD was discovered in the mother's DNA [44]. Her earliest cognitive deficits involved the recent memory, information-processing speed, sequential tracking, and conceptual reasoning. Upon examination, at age 45, she was unable to carry out a conversation, was disoriented for time and space, was echolalic, could not generate speech, and could not recall any information. She needed full assistance for activities of daily living. By age 47, she was mute and bedridden. She died of bronchopneumonia at age 49. On neuropathologic examination, the weight of the fresh brain was 796 g, with the atrophy being most severe at the level of the frontal and temporal lobes. Examination of the cerebral blood vessels reveals mild focal atheromatous changes at the level of the basilar artery and middle cerebral arteries. The fresh brain was hemisected along the mid-sagittal plane. The right cerebral hemisphere, right cerebellar hemisphere and the right half of brain stem were frozen and stored at -80°C. The left hemibrain was fixed for neuropathologic studies. The head of the caudate nucleus, the putamen and the amygdala were atrophic. The white matter of the centrum semiovale was reduced in bulk. No focal lesions were seen in the cortex, subcortical nuclei or white matter. The lateral ventricles were enlarged. The substantia nigra was moderately depigmented. Histologic and immunohistochemical studies revealed nerve cell loss and gliosis throughout the cerebral cortex, neocortex, amygdala, hippocampus, caudate nucleus, putamen, substantia innominata, thalamus. Aβ deposits in the form of neuritic plaques and diffuse plaques were numerous in the cerebral cortex, amygdala, hippocampus, caudate nucleus, putamen, substantia innominata, thalamus, cerebellum, midbrain,

pons, and medulla. Aβ angiopathy was seen in cerebral parenchyma, and leptomeninges. Tau immunopositive NFTs and NTs were numerous in the cerebral cortex, amygdala, hippocampus,substantia innominata, thalamus, midbrain, pons, and medulla. Granulovacuolar degeneration and Hirano bodies were numerous in the hippocampus. Neuropathologic diagnosis was Familial Alzheimer Disease. AD Case 1 had a single nucleotide (G to T) substitution in codon 717 of one allele of the *AβPP* gene. This change results in a phenylalanine for valine amino acid change (V717F). The APOE genotype was ε3/ε3.

Clinical history and pathology of Alzheimer disease Case 2. The patient was a 57-year-old woman with no family history of neurological disease, but with history of head trauma at 10 years of age with a motor vehicle accident. She was a school teacher, who presented with trouble concentrating, poor attention, handwriting changes at age 53. Upon neuropsychological testing, at age 55, the depression scale suggested substantial depressive symptoms. The MMSE score was 21. Word-list learning and delayed recall of word lists were defective. The overall findings were consistent with a dementing illness, most likely AD. The patient had problems with work as her condition deteriorated. Parkinsonian signs were reported in subsequent evaluations at age 56 and 57. A diagnosis of Dementia with Lewy bodies was considered. She was often delusional with agitation and hallucinations. Death occurred at age 57. At the neuropathologic examination, the weight of the fresh brain was 1,009 g. There was moderate atrophy of the frontal, parietal, and temporal lobes. The hippocampus was mildly atrophic, and the lateral ventricles were mildly enlarged. The substantia nigra

and locus coeruleus were moderately depigmented. The fresh brain was hemisected along the mid-sagittal plane. The right cerebral hemisphere, right cerebellar hemisphere and the right half of brain stem were frozen and stored at -80°C. The left hemibrain was fixed for neuropathologic studies. The white matter of the centrum semiovale was reduced in bulk. No focal lesions were seen in the cortex, subcortical nuclei or white matter. The lateral ventricles were enlarged. The substantia nigra was moderately depigmented. Histologic and immunohistochemical studies revealed nerve cell losses and gliosis throughout the cerebral cortex neocortex, amygdala, hippocampus, caudate nucleus, putamen, substantia innominata, and thalamus. Aβ deposits in the form of neuritic plaques and diffuse plaques were numerous in the cerebral cortex, amygdala, hippocampus, caudate nucleus, putamen, substantia innominata, thalamus, and cerebellum. Aβ angiopathy was seen in the cerebral parenchyma and leptomeninges. Tau immunopositive NFTs and NTs were numerous in the cerebral cortex, amygdala, hippocampus,substantia innominata, thalamus, and brainstem. Granulovacuolar degeneration and Hirano bodies were numerous in the hippocampus. Neuropathologic diagnosis was Alzheimer Disease. The APOE genotype was ε3/ε3.

Clinical history and pathology of control case. A 67-year-old man with a past medical history of cardiac arrhythmia died suddenly. Clinical evaluation at the age of 64 years revealed a global CDR of 0. At autopsy, the weight of the fresh whole brain was 1,610 g. The brain was hemisected along the midline. The left hemibrain was frozen and stored at -80°C, whereas the right hemibrain was fixed in formalin. On macroscopic

examination, no pathologic changes were noted. Immunohistochemistry revealed two microscopic foci of diffuse Aβ in the frontal cortex (Thal phase 1). No other cortical deposits of Aβ were noted. No Aβ angiopathy was identified. Occasionally, Tau immunohistochemistry revealed a labelled single isolated neuron in the entorhinal cortex, hippocampus and amygdala. No Lewy bodies or TDP43 positive inclusions were identified.

Supplementary Figure Legends

Figure S1. *Histological and Immunohistochemical features of PrP-CAA (Q160X) and GSS (F198S) compared to AD.* Amyloid and Tau NFTs are fluorescent by Thioflavin S (ThS) staining (a-c). Nerve cell bodies and NTs are decorated by antibodies recognizing phosphorylated Tau, (AT8, d-f), 3 repeat Tau (RD3, g-i) and 4 repeat Tau (RD4, j-l). Sections are from frontal cortex of cases with sporadic late-onset AD (Case 2, a, d, g, and j), PrP-CAA (Q160X) (b, e, h and k) and GSS (F198S) (Case 2, c, f, i and l). Scale bar, 50 nm.

Figure S2. *Characterization of sarkosyl insoluble Tau fraction by dot-blot and immuno-EM.* Representative diagram of the largest human Tau isoform (441 aa). The amino-terminal inserts are labelled N1 and N2. The microtubule-binding repeats are labelled R1 to R4. Epitopes of specific antibodies (abs) used in the study are indicated (a). Dot-blot analysis of the sarkosyl-insoluble fraction from AD, CBD, PiD, CTE, GSS (F198S) and PrP-CAA (Q160X) cases. Samples were analyzed using abs AT8, HT7, BR133, BR134 and BR135 before and after pronase treatment (b). Representative images of immuno-EM of sarkosyl insoluble Tau filaments from the cerebral frontal cortex of PrP-CAA (Q160X) and GSS (F198S) cases without (no pronase) and after (+) pronase treatment. Sarkosyl insoluble Tau filaments from PrP-CAA (Q160X) and GSS (F198S) cases were labelled by BR133, BR134, AT8, HT7 and 4R without pronase treatment, but not by BR135 (c). Scale bar, 50 nm.

Figure S3. *PHF and SF folds in PrP-CAA (Q160X) and GSS (F198S) PHF.* Comparison of the atomic models of PHF (a, d) and SF (b, e), and overlay of both models (c, f) in PrP-CAA (Q160X). Overlay of the atomic models of PHFs from AD (yellow), PrP-CAA (Q160X) (green) and GSS (F198S) (red) (g, h). Rendered view of secondary structure elements of PrP-CAA (Q160X) PHF (i), GSS (F198S) PHF (j) and PrP-CAA (Q160X) SF (k) in three successive rungs.

Figure S4. *Sequence alignment and protofilament interface*. Sequence alignment of the four microtubule-binding repeats (R1–R4) with the observed eight β-strand regions that are part of the fibril cores in AD, PART, CTE, CBD, PiD, PrP-CAA (Q160X) and GSS (F198S) (a). Comparison of inter-protofilament interactions between PrP-CAA (Q160X) PHF, GSS (F198S) PHF, PrP-CAA (Q160X) SF. AD PHF (EMD 3741) and AD SF (EMD 3743) are shown for reference. H-bonds are shown by dotted lines, and some amino acids are marked for reference (b).

Supplementary Table 1. *Cryo-EM data collection, refinement and validation statistics.*

Supplementary Table 2. List of antibodies used in study.

Supplementary Table 3. PTMs of Tau in AD, PrP-CAA (Q160X) and GSS (F198S). PTMs listed are phosphorylation, deamidation, acetylation and ubiquitination in the longest (2N4R) Tau isoform. Number of peptides with the modified residues/total number of peptides containing the amino acid residue are shown.

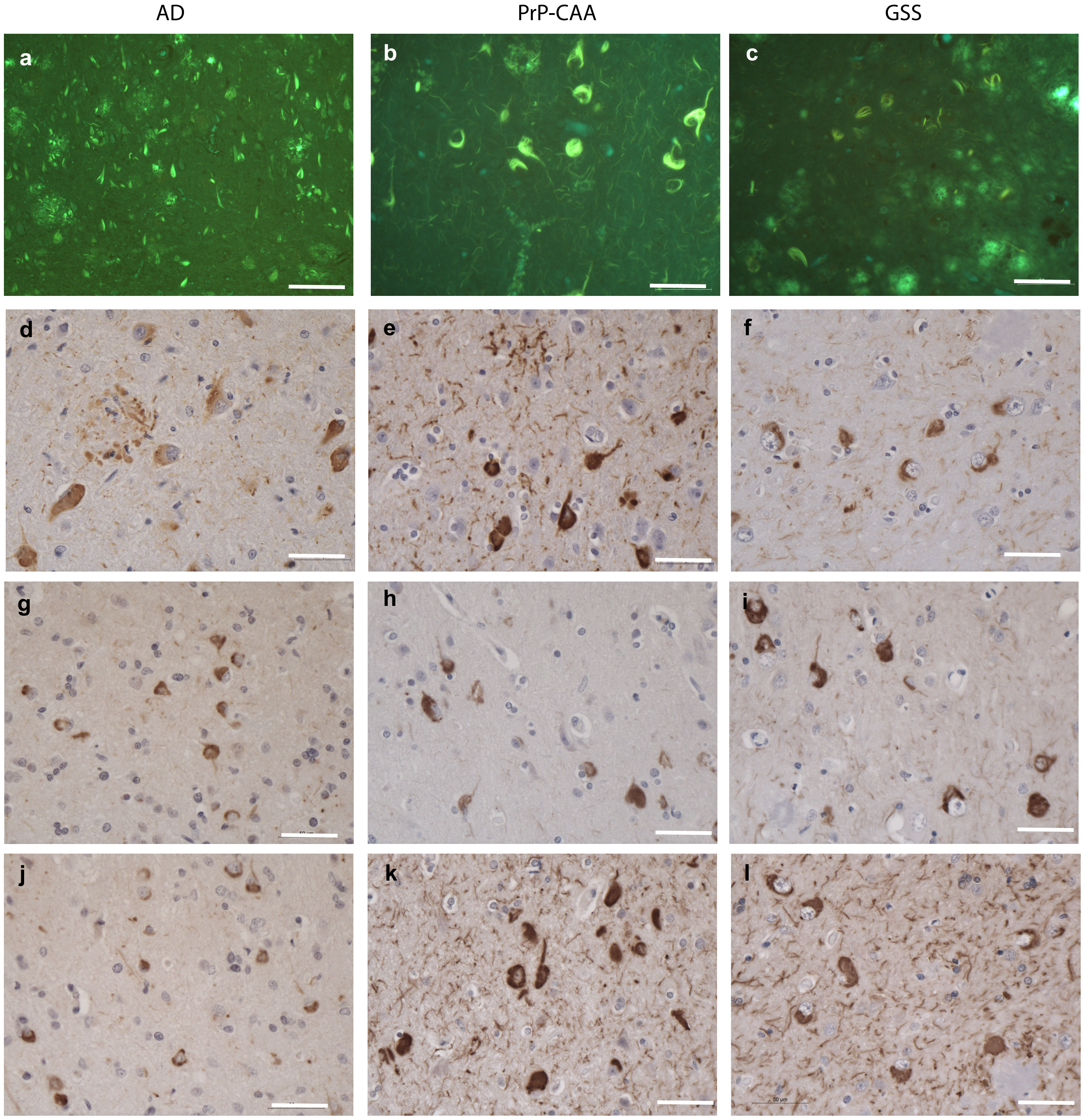

SFigure 1

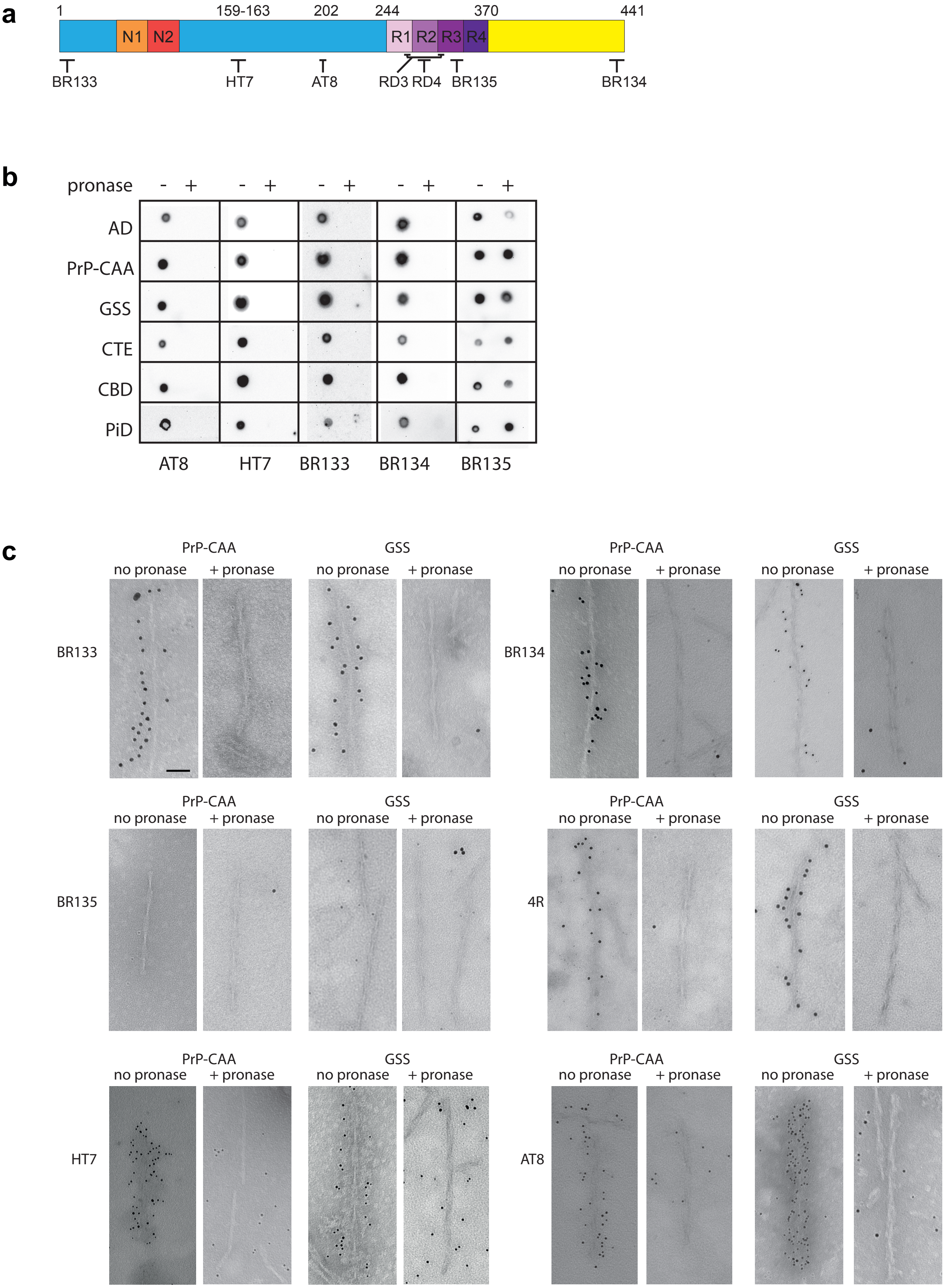
a
1
159-163
202
244
370
441
N1
N2
R1
R2
R3
R4
BR133
HT7
AT8
RD3
RD4
BR135
BR134
b
pronase
- +
AD
PrP-CAA
GSS
CTE
CBD
PiD
AT8
HT7
BR133
BR134
BR135
c
PrP-CAA
GSS
no pronase
+ pronase
BR133
BR134
BR135
4R
HT7
AT8

SFigure 2

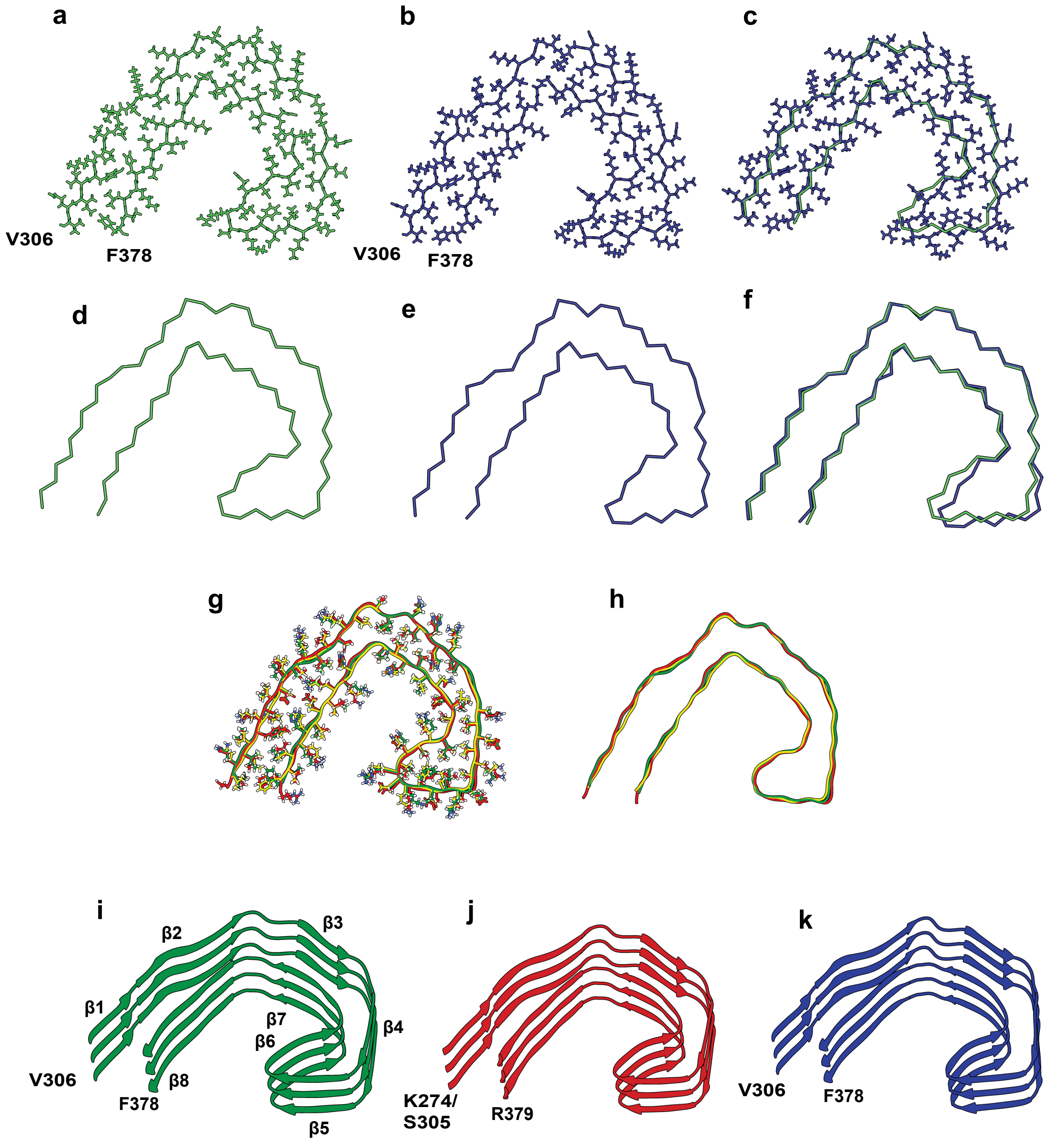
a
b
c
V306
F378
V306
F378
d
e
f
g
h
i
j
k
β1
β2
β3
β4
β5
β6
β7
β8
V306
F378
K274/
S305
R379
V306
F378

SFigure 3

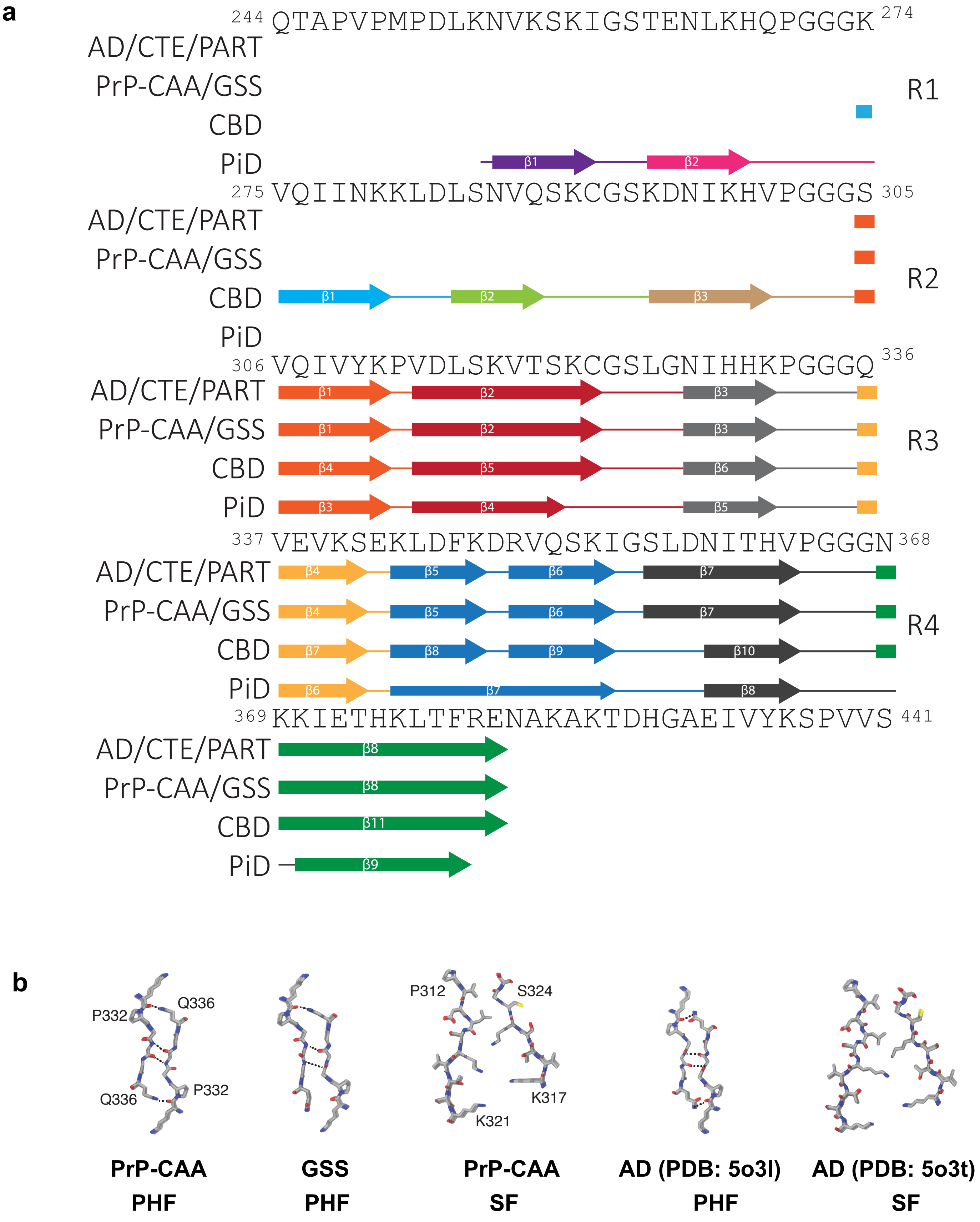
a
244 QTAPVPMPDLKNVKSKIGSTENLKHQPGGGK 274
AD/CTE/PART
PrP-CAA/GSS
CBD
PiD
R1
β1
β2
275 VQIINKKLDLSNVQSKCGSKDNIKHVPGGGS 305
AD/CTE/PART
PrP-CAA/GSS
CBD
PiD
R2
β1
β2
β3
306 VQIVYKPVDLSKVTSKCGSLGNIHHKPGGGQ 336
AD/CTE/PART
PrP-CAA/GSS
CBD
PiD
R3
β1 β2 β3
β1 β2 β3
β4 β5 β6
β3 β4 β5
337 VEVKSEKLDFKDRVQSKIGSLDNITHVPGGGN 368
AD/CTE/PART
PrP-CAA/GSS
CBD
PiD
R4
β4 β5 β6 β7
β4 β5 β6 β7
β7 β8 β9 β10
β6 β7 β8
369 KKIETHKLTFRENAKAKTDHGAEIVYKSPVVS 441
AD/CTE/PART
PrP-CAA/GSS
CBD
PiD
β8
β8
β11
β9
b
P332
Q336
Q336
P332
P312
S324
K317
K321
PrP-CAA
PHF
GSS
PHF
PrP-CAA
SF
AD (PDB: 5o3l)
PHF
AD (PDB: 5o3t)
SF

SFigure 4

Table S1. Cryo-EM data collection, refinement and validation statistics.

Data Collection	PHF (PrP-CAA)	SF (PrP-CAA)	PHF (GSS)
Magnification	x81000	x81000	x81000
Defocus range (μm)	-1.0 to -2.5	-1.0 to -2.5	-1.0 to -3.0
Voltage (kV)	300	300	300
Microscope	Krios	Krios	Krios
Camera	K3	K3	K3
Number of movies collected	2,004	2,004	1,687
Frame exposure time (ms)	62.0	62.0	85.9
Number of frames	50	50	40
Total electron dose ($e^-/Å^2$)	53.35	53.35	59.20
Pixel size (Å)	1.078	1.078	1.078
**Reconstruction**			
Box size (pixel)	300	300	320
Inter-box distance (Å)	15	15	16
Symmetry imposed	C1	C1	C1
Helical rise (Å)	4.80	4.80	4.85
Helical twist (°)	-1.23	-1.23	-1.23
Resolution (Å)	3.0	3. 0	3.2
Map sharpening B-Factor ($Å^2$)	-24	-20	-28
FSC threshold	0.143	0.143	0.143
**Atomic model**			
# unique non-hydrogen atoms	557	557	574
Ramachandran outliers (%)	0.7	1.41	0.0
Ramachandran favored (%)	95.77	92.96	93.15
Rotamer outliers	0.0	0.0	0.0
Cβ deviations	0	0	0
Clashscore	1.67	1.58	0.43
R.m.s.d. angles (°)	0.0229	0.0180	0.0226
R.m.s.d. bonds (Å)	2.00	1.73	1.73
MolProbity Score	1.21	1.35	1.09
iFSC	0.573	0.591	0.566

Table S2. Antibody list

Antigen	Host	Antibody	Source
Phospho-Tau (pSer202 and pThr205)	Mouse	AT8	Thermo Fisher
Phospho-Tau (pSer396 and pSer404)	Mouse	PHF1	P. Davies, Albert Einstein College of Medicine; New York; USA
3-repeat Tau	Mouse	RD3	Millipore
4-repeat Tau	Mouse	RD4	Millipore
4-repeat Tau	Rabbit	4R	Cosmo Bio
Tau (human)	Mouse	HT7	Thermo Fisher
Tau (N terminal)	Rabbit	BR133	M. Goedert, Medical Research Council Laboratory of Molecular Biology
Tau (C terminal)	Rabbit	BR134	M. Goedert, Medical Research Council Laboratory of Molecular Biology
Tau (R3 terminus)	Rabbit	BR135	M. Goedert, Medical Research Council Laboratory of Molecular Biology
PrP 95-108	Rabbit	PrP 95-108	B. Ghetti, Indiana University School of Medicine
PrP 109-122	Mouse	3F4	Millipore
Amyloid beta (N-terminal)	Mouse	NAB228	Thermo Fisher

Table S3. Quantification of mass spectrometry peptides with PTMs

Phosphorylation

	AD	PrP-CAA Q160X	GSS F198S
T30	0/15	0/12	0/5
T39	0/17	0/11	0/5
S46	0/10	0/5	0/2
T50	0/10	0/5	0/2
T52	0/10	0/5	0/2
T153	2/7	2/4	2/4
T175	5/37	3/20	3/25
T181	12/38	8/19	8/17
S184	2/40	0/19	0/17
S185	0/41	1/19	0/17
S191	1/27	0/10	0/7
S195	0/9	0/6	0/3
S198	0/9	0/7	0/3
S199	1/9	2/7	0/3
S202	2/10	5/10	1/3
T205	0/10	0/10	0/3
S208	0/10	0/10	0/3
S210	0/17	0/7	0/3
T212	1/22	1/14	0/10
S214	5/32	1/15	0/10
T217	15/32	5/15	3/10
T220	1/32	0/15	0/10
T231	23/35	18/30	14/19
S235	12/33	12/29	8/19
S237	0/33	0/29	0/19
S238	4/33	6/29	2/19
S241	0/32	0/23	0/15
T245	0/77	0/49	0/35
S258	0/15	0/13	0/7
S262	9/43	12/31	7/18
S285	4/40	2/22	1/13
S289	4/40	3022	1/12
S305	1/24	1/26	0/10
S316	0/29	0/43	0/13
S320	0/3	1/4	-
T263	1/43	0/32	0/22
S324	2/43	1/25	0/20
S352	0/10	0/9	0/3
S356	2/40	2/36	0/13
T361	0/43	0/38	0/13
T386	0/17	0/21	0/11
S396	9/20	10/20	3/9
S400	5/20	6/20	1/9
T403	1/20	0/18	0/9
S404	5/19	4/18	1/9
S409	0/15	0/10	0/4
S412	0/15	0/10	0/4
S413	0/15	0/10	0/4
T414	0/15	0/10	0/4
S416	0/15	0/10	0/4
S422	2/17	2/12	0/5
T427	0/25	0/12	0/6
S433	1/21	1/27	0/6

Deamidation

	AD	PrP-CAA Q160X	GSS F198S
Q6	4/37	1/25	1/22
Q26	0/17	0/12	0/5
Q33	2/17	0/11	0/5
Q35	1/17	0/11	0/5
Q49	1/10	0/5	0/2
R155	2/7	0/4	0/4
Q162	3/18	1/8	0/8
Q165	3/33	0/18	2/22
Q167	16/33	8/18	10/22
R170	1/34	1/18	1/22
R194	1/26	0/7	0/8
R209	1/10	0/10	0/3
R242	3/33	1/24	1/15
Q244	9/76	4/49	5/35
N255	16/39	14/25	9/16
N265	16/43	14/32	9/21
Q269	1/31	0/20	0/10
N279	8/15	3/3	0/1
N286	12/40	5/21	6/12

	AD	PrP-CAA Q160X	GSS F198S
Q288	3/40	0/21	0/12
N296	2/8	1/4	-
Q307	1/29	3/39	1/13
R327	6/44	2/26	4/20
R349	1/8	0/8	0/6
Q351	0/10	1/8	0/3
N359	2/43	0/38	0/13
N368	4/43	0/38	3/13
R379	1/14	0/10	0/6
N381	4/11	3/6	3/5
N410	0/15	2/10	0/4
Q424	1/17	3/12	0/5
**Acetylation**			
M11	3/45	4/29	0/23
K234	0/35	0/30	0/19
K240	0/33	0/29	0/19
K254	1/78	0/56	0/35
N255	0/39	0/25	1/16
K257	0/39	0/18	0/16
K259	0/14	0/13	0/7
K267	0/43	0/32	0/21
K274	0/14	0/20	0/5
K280	0/17	0/4	0/1
K281	0/33	0/14	0/8
K290	1/40	0/21	0/12
K311	0/30	2/43	1/13
K317	0/29	0/41	0/13
K321	0/3	0/4	0/3
K331	2/45	0/28	0/20
K343	0/36	0/7	0/2
K353	1/13	0/10	0/3
K369	1/42	1/36	0/13
K370	0/25	0/24	0/13
K375	0/8	0/8	0/2
K383	0/11	0/6	0/5
**Ubiquitination**			
K150	0/3	0/3	0/1

	AD	PrP-CAA Q160X	GSS F198S
K174	0/40	0/25	0/28
K180	0/35	0/23	0/27
K190	0/41	0/19	0/17
K225	0/17	0/13	0/10
K234	0/35	0/30	0/19
K240	0/33	0/29	0/19
K254	6/78	0/56	135
K257	0/39	0/18	0/166
K259	0/14	0/13	0/7
K267	2/43	0/32	2/22
K274	4/14	0/20	0/5
K280	0/17	0/4	0/1
K281	1/32	0/14	0/8
K290	0/40	0/21	0/12
K298	0/9	0/6	-
K311	2/30	3/43	2/13
K317	0/29	2/41	1/13
K343	0/36	0/7	0/2
K353	0/13	0/10	0/3
K369	0/42	0/36	0/13
K370	0/25	0/24	0/13
K375	0/8	0/8	0/2
K385	0/5	0/8	0/4
K395	0/16	0/20	0/11
